# Coordinate regulation of long non-coding RNAs and protein-coding genes in germ-free mice

**DOI:** 10.1186/s12864-018-5235-3

**Published:** 2018-11-21

**Authors:** Joseph Dempsey, Angela Zhang, Julia Yue Cui

**Affiliations:** Department of Environmental and Occupational Health Sciences, University of Washington, 4225 Roosevelt Way NE, Seattle, WA 98105 USA

**Keywords:** lncRNAs, Gut microbiota, Mouse, Tissue distribution

## Abstract

**Background:**

Long non-coding RNAs (lncRNAs) are increasingly recognized as regulators of tissue-specific cellular functions and have been shown to regulate transcriptional and translational processes, acting as signals, decoys, guides, and scaffolds. It has been suggested that some lncRNAs act in *cis* to regulate the expression of neighboring protein-coding genes (PCGs) in a mechanism that fine-tunes gene expression. Gut microbiome is increasingly recognized as a regulator of development, inflammation, host metabolic processes, and xenobiotic metabolism. However, there is little known regarding whether the gut microbiome modulates lncRNA gene expression in various host metabolic organs. The goals of this study were to 1) characterize the tissue-specific expression of lncRNAs and 2) identify and annotate lncRNAs differentially regulated in the absence of gut microbiome.

**Results:**

Total RNA was isolated from various tissues (liver, duodenum, jejunum, ileum, colon, brown adipose tissue, white adipose tissue, and skeletal muscle) from adult male conventional and germ-free mice (*n* = 3 per group). RNA-Seq was conducted and reads were mapped to the mouse reference genome (mm10) using HISAT. Transcript abundance and differential expression was determined with Cufflinks using the reference databases NONCODE 2016 for lncRNAs and UCSC mm10 for PCGs. Although the constitutive expression of lncRNAs was ubiquitous within the enterohepatic (liver and intestine) and the peripheral metabolic tissues (fat and muscle) in conventional mice, differential expression of lncRNAs by lack of gut microbiota was highly tissue specific. Interestingly, the majority of gut microbiota-regulated lncRNAs were in jejunum. Most lncRNAs were co-regulated with neighboring PCGs. STRING analysis showed that differentially expressed PCGs in proximity to lncRNAs form tissue-specific networks, suggesting that lncRNAs may interact with gut microbiota/microbial metabolites to regulate tissue-specific functions.

**Conclusions:**

This study is among the first to demonstrate that gut microbiota critically regulates the expression of lncRNAs not only locally in intestine but also remotely in other metabolic organs, suggesting that common transcriptional machinery may be shared to transcribe lncRNA-PCG pairs, and lncRNAs may interact with PCGs to regulate tissue-specific pathways.

**Electronic supplementary material:**

The online version of this article (10.1186/s12864-018-5235-3) contains supplementary material, which is available to authorized users.

## Background

With the advent of transcriptomic studies, it was revealed that only 2% of the genome has protein-coding capacity [1, 2], and the vast majority of transcripts that do not have protein coding capacity are called non-coding RNAs (ncRNAs). This broad category of functional RNA transcripts is divided into two major groups: small ncRNAs which are less than 200 nucleotides, including microRNAs, short interfering RNAs and piwi-interacting RNAs; as well as long noncoding RNAs (lncRNAs) that are longer than 200 nucleotides. LncRNAs may have their own promoter regions, DNA binding motifs, and transcription factors [3], and computational analyses suggest that the transcription of lncRNAs may occur independently and influence the expression of protein-coding genes (PCGs) [3, 4]. LncRNAs are increasingly recognized as regulators of cellular functions and have been shown to regulate transcriptional and translational processes, acting as signals, decoys, guides, and scaffolds [5–8]. For example, the lncRNA HOTAIR from the homeobox (HOX) C cluster is an epigenetic-protein scaffold with the Polycomb Repressive Complex 2 (PRC2) and is required for PRC2 tri-methylation of histone H3 lysine 27 (H3K27Me3) at the HOXD locus for gene silencing [9]. Conversely, the lncRNA HOTTIP, which is found downstream of the HOXA cluster, interacts with the WD repeat domain (WDR) 5 and mixed-lineage leukemia (MLL) to drive H3K4Me3 for gene activation [5]. LncRNAs, such as HOTAIR, HOTTIP, Xist and Air, also regulate chromatin architecture and gene transcription in *cis* by interacting with epigenetic complexes [5, 10–12]. Conversely, other lncRNAs, such as LincRNA-p21 and HOTAIR in cancer cells, can mediate chromatin architecture and gene expression in *trans*; for example, LincRNA-p21 assists a gene-silencing complex to bypass the upstream regulator p53 for apoptosis [5, 13]. Collectively, these initial studies identified lncRNAs as necessary moderators of chromatin architecture and gene expression through interactions with PCGs.

Furthermore, it has been suggested that some lncRNAs may affect the expression of neighboring PCGs [14, 15]. The expression of lncRNAs and proximal PCGs are more correlated than random gene pairs [16]. This suggests that some lncRNAs act in *cis* to regulate the expression of PCGs. Indeed, as demonstrated in zebrafish, the lncRNA slincR is regulated by the transcription factor aryl hydrocarbon receptor 2 (AHR2) and acts as an intermediate modulator between AHR2 activation and decreased expression of the proximal PCG sex-determining region Y-box (sox) 9b [17]. Computational analyses identified increased lncRNA-mRNA interactions when stratifying by tissue-specific expression patterns, indicating a tissue-specific regulatory pattern for lncRNAs [18]. Identification of PCG-lncRNA pairs and further mechanistic validation is necessary to assess the regulatory functions of lncRNAs.

According to NONCODEV5 database, which hosts the most complete collection of annotated lncRNAs across 17 species, there are 172,216 human and 131,697 mouse lncRNA transcripts [19]. In humans, only 11–29% of lncRNAs are ubiquitously expressed in all tissues compared to 65% of PCGs [2, 4, 16]. Indeed, a study comparing human pancreatic islet cells to 16 non-pancreatic RNA-Seq datasets found that over 9% of the annotated lncRNAs were islet specific [20]; the group also found aberrant lncRNA expression from donors with Type 2 Diabetes, indicating that lncRNA regulation is essential to a healthy system. In an extensive study that evaluated lncRNAs in over 7000 RNA-Seq libraries from 25 studies, about 3900 lncRNAs overlapped with disease-associated single nucleotide polymorphisms. This suggests a regulatory and functional role of lncRNAs that influences overall health [21].

Gut microbiome is increasingly recognized as a regulator of development, inflammation, host metabolic processes, and xenobiotic metabolism [22–26]. In liver, there are profound differences in the expression patterns of xenobiotic-processing genes between conventional (CV, i.e. with gut microbiome) and germ-free (GF, i.e. without gut microbiome) mice [27–31]. In addition, microbial metabolites such as secondary bile acids and short chain fatty acids act as signaling molecules to metabolic organs, such as brown adipose tissue (BAT) and white adipose tissue (WAT), and brain [32–35]. However, there is little information regarding whether the gut microbiome modulates lncRNA expression in various host metabolic organs.

Therefore, the goal of the present study was to use GF mice and RNA-Seq to 1) characterize the tissue-specific expression of lncRNAs in liver, duodenum, jejunum, ileum, colon, BAT, WAT, and skeletal muscle, 2) identify lncRNAs differentially regulated by the absence of gut microbiota, and 3) unveil the functional networks of gut microbiota-regulated lncRNA-PCG pairs. This study is among the first to characterize the regulation of lncRNAs and PCGs in target organs of the gut microbiome, and is among the first to unveil the potential PCG targets by lncRNAs in a tissue-specific manner.

## Results

The present study used RNA-Seq to determine the effect of the absence of gut microbiota on the transcriptional regulation of lncRNAs and the paired PCGs in eight organs from CV and GF mice. Approximately 34 to 89 million reads per sample with 92 to 97% of the reads were mapped uniquely to the mouse reference genome (NCBI GRCm38/mm10). This results in 34 to 86 million uniquely mapped reads (Additional file 1: Table S1).

### Distribution of expressed lncRNAs in control CV mice

**Fig. 1:**
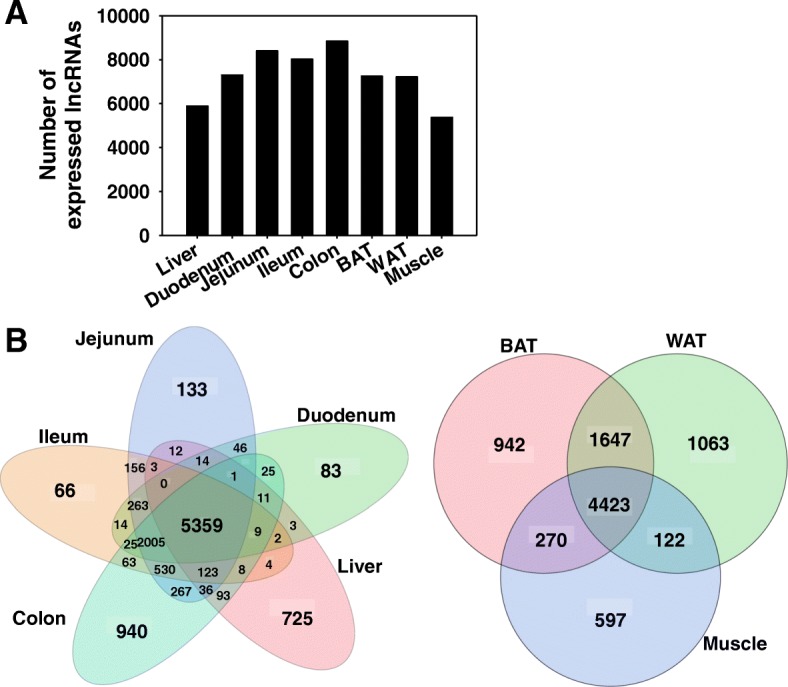
Distribution of expressed lncRNAs in eight organs of CV mice. (**a**) Bar chart showing the number of lncRNAs expressed (average FPKM > 1 in either CV or GF conditions) in liver, duodenum, jejunum, ileum, colon, BAT, WAT, and muscle. (**b**) Venn diagram showing the number of expressed lncRNAs that were commonly or uniquely expressed in each organ. Venn diagram was generated using JMP Genomics

Among the 125,680 annotated lncRNAs in the mouse NONCODE 2016 reference database, 11,841 lncRNAs were expressed in at least one organ (threshold: average fragments per kilobase of exon per million reads mapped [FPKM] > 1 in at least one tissue of CV mice). Intestine had the largest numbers of expressed lncRNAs (7302 in duodenum, 8396 in jejunum, 8018 in ileum, 8836 in colon), followed by BAT (7246), WAT (7214), liver (5886), and muscle (5374) (Fig. 1a). To identify how many lncRNAs were tissue-specific and how many were universally expressed, Venn diagrams of the major tissues involved in enterohepatic circulation, namely liver, duodenum, jejunum, ileum, and colon, and the peripheral metabolic tissues, namely BAT, WAT, and muscle, were generated (Fig. 1b). Within the liver and various intestine sections, there were 5359 commonly expressed lncRNAs. Colon had the largest number of uniquely expressed lncRNAs (940), followed by liver (725), jejunum (133), duodenum (83), and ileum (66). For the peripheral metabolic organs, 4423 lncRNAs were commonly expressed in BAT, WAT, and muscle. WAT had the largest number of uniquely expressed lncRNAs (1063), followed by BAT (942) and muscle (597).

**Fig. 2:**
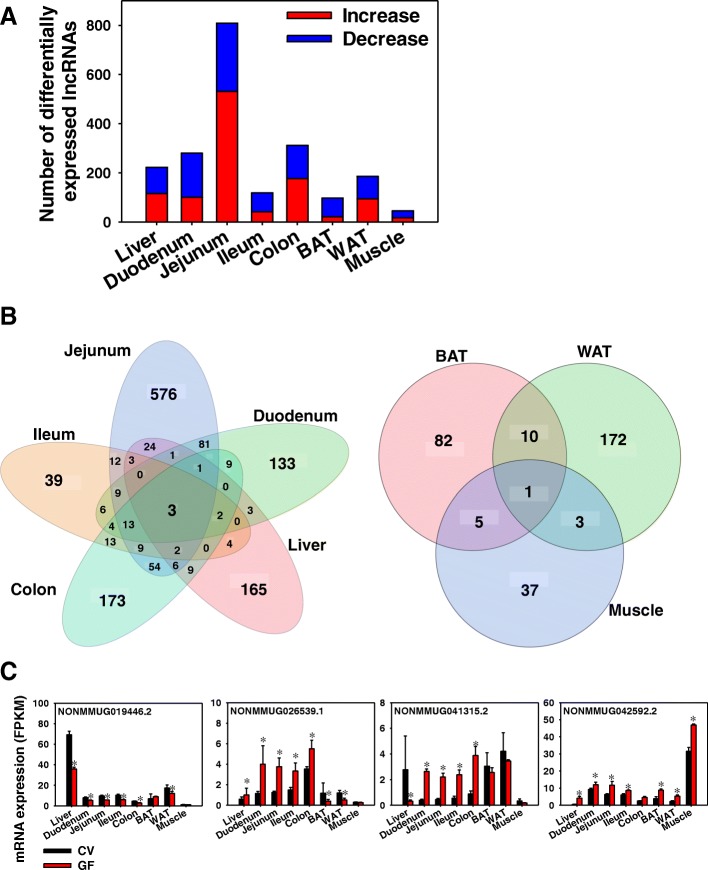
Distribution of differentially expressed lncRNAs in eight organs between CV and GF mice. (**a**) Bar chart showing the number of expressed lncRNAs (average FPKM > 1 in either CV or GF conditions) that were differentially regulated by lack of gut microbiota in liver, duodenum, jejunum, ileum, colon, BAT, WAT, and muscle (FDR-BH < 0.05). (**b**) Venn diagram showing the number of differentially expressed lncRNAs by lack of gut microbiota in each organ. Liver and various sections of intestine that are proximal to gut micrboiome were grouped together; whereas the energy metabolism related organs (BAT, WAT, and muscle) that are distal to gut microbiota were grouped together. Venn diagram was generated using JMP Genomics

To determine the effect of the absence of gut microbiota on the hepatic expression of lncRNAs, Cuffdiff was performed in expressed lncRNAs between CV and GF mice in the same organ (threshold: average FPKM > 1 in CV or GF mice and Benjamini-Hochberg adjusted false discovery rate [FDR-BH] < 0.05) (Fig. 2a). In liver, the lack of gut microbiota up-regulated 116 lncRNAs and down-regulated 107 lncRNAs. In duodenum, the lack of gut microbiota up-regulated 101 lncRNAs and down-regulated 179 lncRNAs. Jejunum had the largest number of differentially regulated lncRNAs as a result of lack of gut microbiota, including 531 up-regulated lncRNAs and 278 down-regulated lncRNAs. In ileum, the lack of gut microbiota up-regulated 42 lncRNAs and down-regulated 77 lncRNAs. In colon, the lack of gut microbiota up-regulated 177 lncRNAs and down-regulated 135 lncRNAs. In BAT, the lack of gut microbiota up-regulated 21 lncRNAs and down-regulated 77 lncRNAs. In WAT, the lack of gut microbiota up-regulated 95 lncRNAs and down-regulated 91 lncRNAs. In muscle, which had the fewest differentially regulated lncRNAs, the lack of gut microbiota up-regulated 17 lncRNAs and down-regulated 29 lncRNAs.

Two-way hierarchical clustering dendrograms of the differentially regulated lncRNAs per organ are shown in Additional file 2: Figure S1. As shown in Fig. 2b, the majority of differentially regulated lncRNAs were unique per tissue type. Among the enterohepatic tissues, jejunum had the most uniquely differentially regulated lncRNAs by lack of gut microbiota (576), followed by colon (173), liver (165), duodenum (133), and ileum (39). There were three lncRNAs that were differentially regulated in all five organs, namely NONMMUG019446.2, NONMMUG026539.1, and NONMMUG041315.2 (Fig. 2c). In the peripheral metabolic tissues, fewer lncRNAs were differentially regulated by lack of gut microbiota as compared to the enterohepatic tissues. WAT had the most differentially regulated lncRNAs (172), followed by BAT (82) and muscle (37). The lncRNA NONMMUG042592.2 was differentially increased in all three peripheral metabolic organs (Fig. 2c).

### Genomic annotation of lncRNAs differentially regulated by lack of gut microbiota

**Fig. 3:**
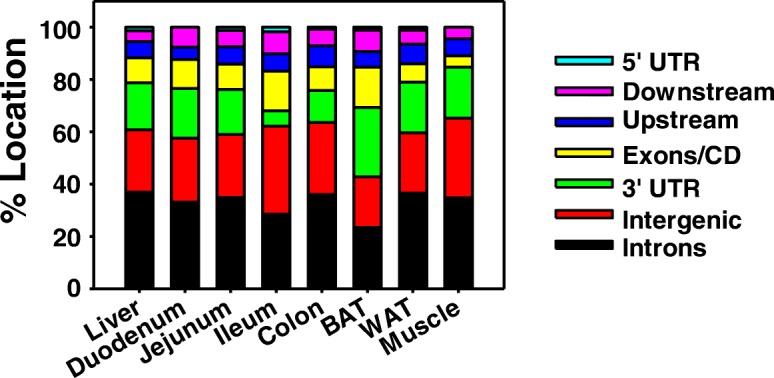
Genomic annotation of differentially expressed lncRNAs detected. A stacked bar chart showing the percent of differentially expressed lncRNAs (average FPKM > 1 in either CV or GF conditions, and FDR-BH < 0.05 between CV and GF conditions) annotated to each genomic region relative to PCGs, including up to 5 kb upstream of transcription start site (TSS), intronic, exonic, 5′-untranslated region (UTR), 3’-UTR, and up to 1 kb downstream of transcriptional termination site (TTS). LncRNAs that were not identified to these locations were considered intergenic. Chromosome coordinates of these differentially regulated lncRNAs in each organ were analyzed using PAVIS

To determine the genomic locations of differentially expressed lncRNAs by the lack of gut microbiota in each organ, we used the web-based tool peak annotation and visualization (PAVIS) to annotate these lncRNAs (Fig. 3). The lncRNAs were considered proximal to PCGs if they are located within 5 kb upstream or 1 kb downstream of the nearest PCG locus. The lncRNAs outside this range were considered intergenic. In general, across all eight organs the majority of differentially regulated lncRNAs were mapped to the intronic regions (35.4%) of PCGs and intergenic regions (24.4%), followed by 3′- untranslated region (UTR; 16.0%). Moderate portions of lncRNAs were mapped to exonic (10.3%), upstream (6.8%), and downstream regions (6.1%), and a small fraction of lncRNAs were mapped to the 5’-UTR regions (1.1%).

### Network interactions of lncRNA-PCG pairs differentially regulated by lack of gut microbiota

The regulation of intergenic lncRNAs in the eight target organs by the absence of gut microbiota is shown in Additional file 1: Tables S3-S10. In general, jejunum had the largest numbers of differentially regulated intergenic lncRNAs (118 up-regulated and 78 down-regulated in the absence of gut microbiota), followed by colon (43 up- and 44 down-regulated), duodenum (22 up- and 47 down-regulated), liver (21 up- and 33 down-regulated), WAT (26 up- and 17 down-regulated), and ileum (16 up- and 24 down-regulated), whereas muscle (3 up- and 11 down-regulated) and BAT (2 up- and 9 down-regulated) had the fewest differentially regulated intergenic lncRNAs. Regarding the lncRNAs that are present near PCGs, to predict the potential functions of the differentially regulated lncRNAs by lack of gut microbiota for each organ, we paired the differentially regulated lncRNAs with their neighboring PCGs by three criteria: 1) the lncRNA transcript is annotated within 5 kb upstream of the transcription start site (TSS) and 1 kb downstream of the transcriptional termination site (TTS) of a PCG; 2) both the lncRNA and the PCG are expressed under CV or GF conditions (FPKM > 1); and 3) both the lncRNA and the PCG are differentially regulated by lack of gut microbiota (FDR-BH < 0.05). Another key assumption was that the lncRNAs that are differentially regulated together with neighboring PCGs by lack of gut microbiota will influence the expression of these PCGs more than lncRNAs produced from distal regions. Using these criteria, PCGs that were differentially regulated by germ free conditions, as well as lncRNAs that were differentially regulated by germ free conditions, were retrieved independently per organ for step 1. In step 2, all differentially regulated lncRNAs that had a differentially regulated PCG in the same neighborhood were defined as lncRNA-PCG pairs and were kept for further analysis. The gene symbols, loci, and expression values of the lncRNA-PCG pairs is listed in Additional file 1: Table S2 for each tissue. As shown in Table 1, jejunum had the largest numbers of lncRNA-PCG pairs (358), followed by colon (122), liver (105), BAT (56), duodenum (52), WAT (39), ileum (32), and muscle (16). Interestingly, nearly all lncRNA-PCG pairs in all organs were co-regulated by lack of gut microbiota (i.e. the lncRNA and PCG in each pair were both up-regulated or both down-regulated), suggesting that they may share the same transcription machinery in a particular organ following changes in gut microbiota. The only two exceptions are: 1) in liver, the lncRNA NONMMUG068817.1 was up-regulated whereas the paired PCG glutathione synthetase (Gss) was down-regulated by lack of gut microbiota, and both of them are transcribed from the Crick strand; 2) in jejunum, the lncRNA NONMMUG042631.1, which is transcribed from the Watson strand, was up-regulated by lack of gut microbiota, whereas the paired PCG pleckstrin homology like domain, family B, member 1 (Phldb1), which is transcribed from the Crick strand, was down-regulated by lack of gut microbiota.

**Table 1:** Numbers of differentially regulated lncRNA-PCG pairs

Organ	Total lncRNA-PCG pairs	Both up-regulated	Both down-regulated	LncRNA up-regulated and PCG down-regulated
Liver	105	58	46	1
Duodenum	52	36	16	0
Jejunum	358	255	102	1
Ileum	32	11	21	0
Colon	122	76	46	0
BAT	56	9	47	0
WAT	39	22	17	0
Muscle	16	8	8	0

To predict the interactions of the differentially regulated PCGs that paired with a lncRNA, we used Search Tool for the Retrieval of Interacting Genes/Proteins (STRING) to generate protein-protein association networks [36]. For these networks, only the nodes (proteins) that are connected (edges) in a network are shown.

#### Liver

**Fig. 4:**
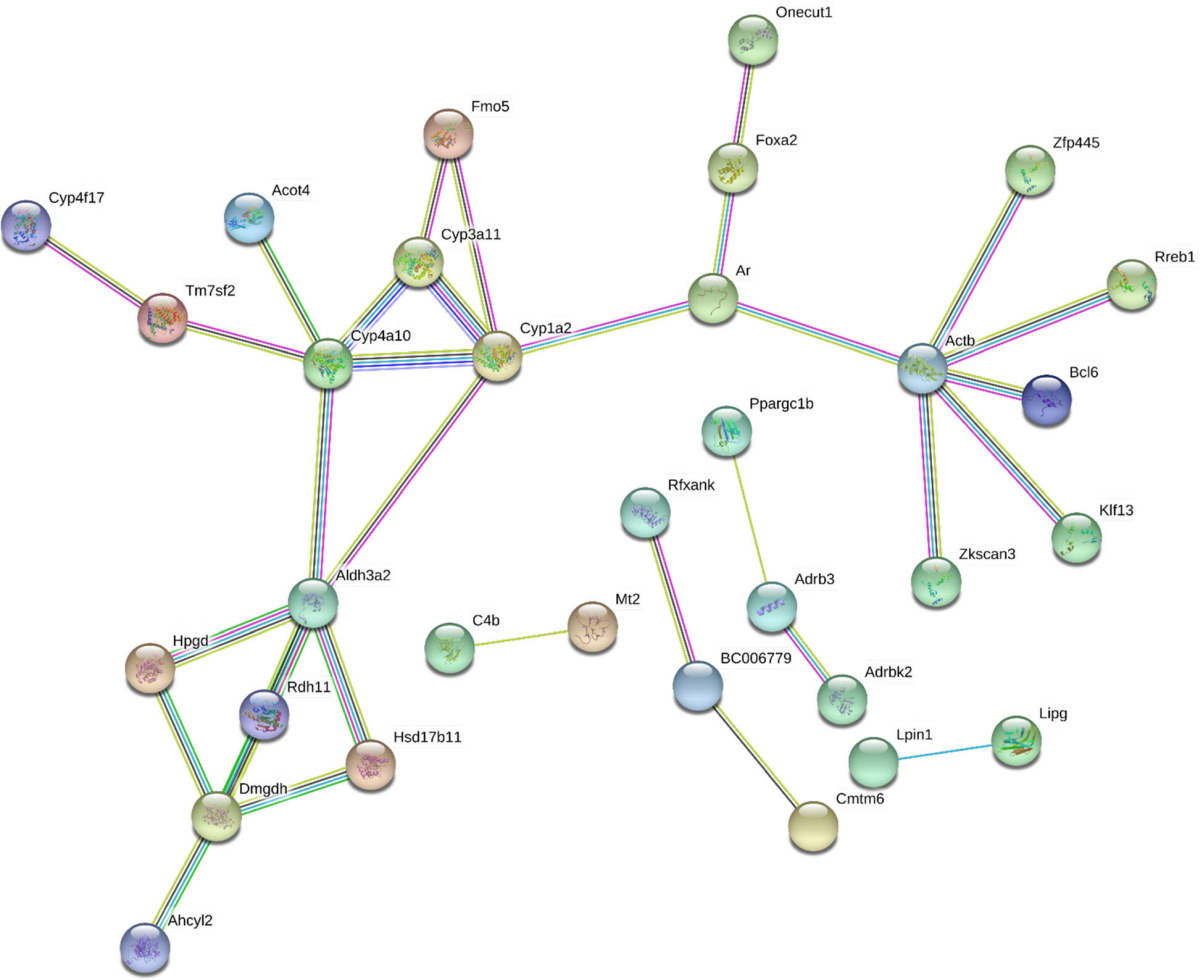
Pathway analysis of PCGs paired with lncRNAs that were differentially regulated by lack of gut microbiota in livers of CV and GF mice. The lncRNA-PCG pairs in liver between CV and GF mice were subjected to STRING analysis using the default settings. The connected nodes are shown

As shown in Fig. 4, in liver of GF mice, 33 edges were present from 94 unique PCGs with a protein-protein interaction (PPI) enrichment *p*-value of 0.0346. For example, many Phase-I drug-metabolizing enzymes, such as the P450 family members (Cyp1a2, Cyp3a11, Cyp4a10, and Cyp4f17), the flavin containing monooxygenase 5 (Fmo5), aldehyde dehydrogenase 3a2 (Aldh3a2), and hydroxysteroid 17β dehydrogenase (Hsd17b11), were all paired with lncRNAs in liver (Fig. 4). The metal-binding protein metallothionein 2 (Mt2), which is induced under many stress responses, as well as several transcription factors such as androgen receptor (Ar), Onecut1, Foxa2, and Ppargc1b (also known as PGC1β), were all paired with lncRNAs in liver. Enriched KEGG pathways included metabolic pathways (Pathway ID: 01100; FDR = 0.008) and retinol metabolism (Pathway ID: 00830; FDR = 0.0495). Therefore, lncRNAs that were co-regulated with the paired PCGs may contribute to the regulation of these xenobiotic and intermediary metabolic pathways in liver.

**Fig. 5:**
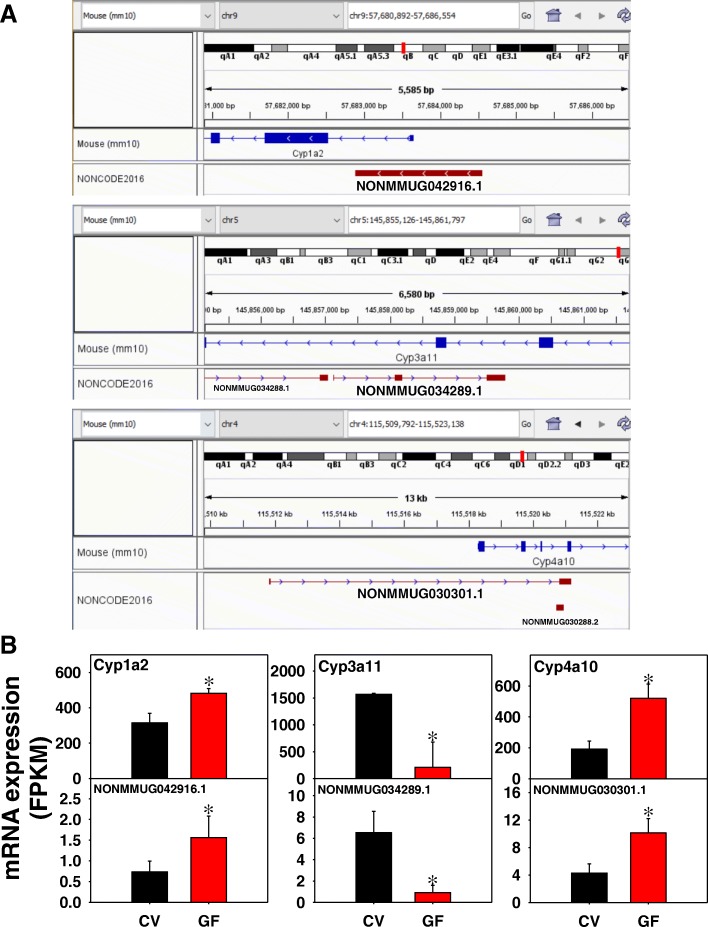
Genomic locations (**a**) and gene expression (**b**) of examples of lncRNA-PCG pairs that were differentially regulated by lack of gut microbiota in livers of CV and GF mice. Drug metabolizing enzymes Cyp1a2, Cyp3a11, and Cyp4a10 are shown. Expression of lncRNAs and paired PCGs were plotted using mean FPKM ± S.E., and asterisks (*) indicate statistically significant differences between enterotypes of mice (FDR-BH < 0.05)

Examples of the genomic locations and expression of the lncRNA-PCG pairs are shown in Fig. 5. Because liver is a major organ for the oxidative metabolism of many drugs and other xenobiotics, P450 family members are shown. The lncRNA NONMMUG042916.1 is located on chromosome 9 and is transcribed upstream and overlaps with the 5’-UTR of Cyp1a2, which generally oxidizes planar, polycyclic aromatic hydrocarbons (Fig. 5a). Both NONMMUG042916.1 and Cyp1a2 are transcribed from the Crick strand and were both up-regulated by the lack of gut microbiota (Fig. 5b). The lncRNA NONMMUG034289.1 is located on chromosome 5 and is transcribed from the intronic regions of Cyp3a11, which is a major P450 family member with promiscuous activity toward many xenobiotic substrates. Both NONMMUG034289.1 and Cyp3a11 were down-regulated by lack of gut microbiota, despite being transcribed from opposite strands, suggesting that they are regulated by two coordinated transcription machineries or that the transcription complex may hop strands to synchronize the transcription of two distinct transcripts (Fig. 5a and b). NONMMUG030301.1 is located on chromosome 4 and is transcribed from the 5’-UTR to the intronic region of its neighboring PCG Cyp4a10 (a fatty acid oxidation enzyme) (Fig. 5a), and they were both up-regulated by lack of gut microbiota (Fig. 5b). The expression and co-regulation of other lncRNA-PCG pairs involved in cholesterol biosynthesis and transport, drug metabolism and oxidative stress, and nucleoside transport are included in Additional file 2: Figure S2.

#### Duodenum

**Fig. 6:**
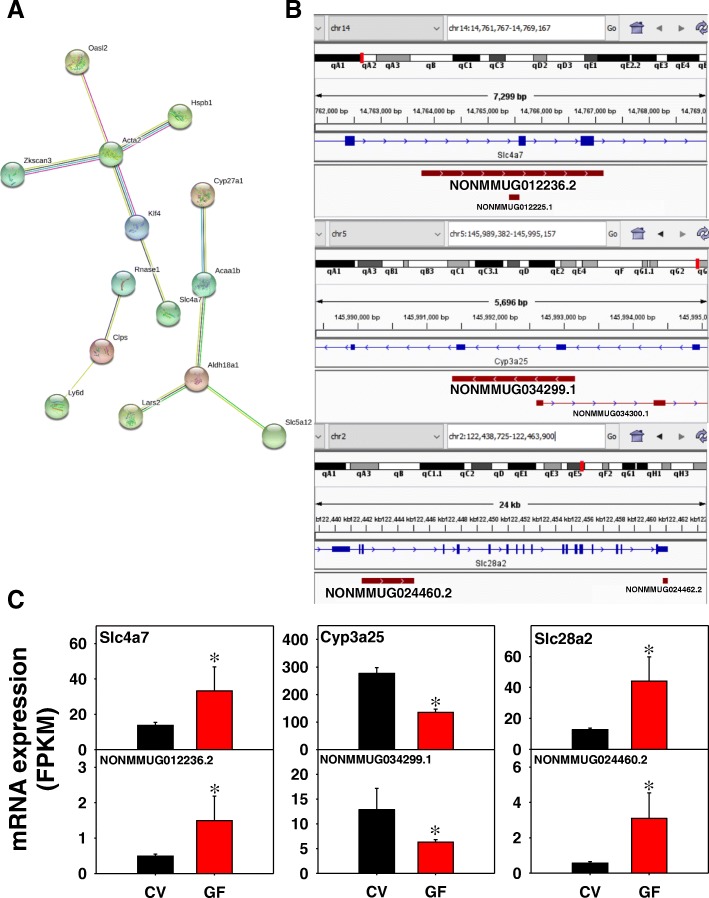
Pathway analysis (**a**), as well as genomic locations (**b**) and gene expression (**c**) of examples of lncRNA-PCG pairs that were differentially regulated by lack of gut microbiota in duodenum of CV and GF mice. (**a**) PCGs paired with lncRNAs in duodenum between CV and GF mice were subjected to STRING analysis using the default settings. The connected nodes are shown. (**b**) and (**c**) The sodium bicarbonate transporter solute carrier (Slc) 4a7, the phase-I oxidation enzyme Cyp3a25, and the sodium-dependent purine transporter Slc28a2 are shown. Expression of lncRNAs and paired PCGs were plotted using mean FPKM ± S.E., and asterisks (*) indicate statistically significant differences between enterotypes of mice (FDR-BH < 0.05)

As shown in Fig. 6a, in duodenum of GF mice, 11 edges were present from 49 unique PCGs with a PPI enrichment *p*-value of 0.0523. Examples of the lncRNA-PCG pairs are shown in Fig. 6b and c. The lncRNA NONMMUG012236.2 on chromosome 14 is transcribed from the intronic region of the sodium and bicarbonate electroneutral cotransporter solute carrier family 4 member 7 (Slc4a7/NBCn1). Both NONMMUG012236.2 and Slc4a7 are transcribed from the Watson strand and were up-regulated in the absence of gut microbiota (Fig. 6c). Another intronic lncRNA, namely NONMMUG034299.1, is transcribed from the Crick strand along with the Phase-I oxidation enzyme Cyp3a25, and both transcripts were down-regulated by the lack of gut microbiota. Interestingly, a second lncRNA NONMMUG034300.1 transcribed from the opposite strand overlaps with NONMMUG034299.1 and Cyp3a25, but was not significantly expressed in duodenum in either CV or GF conditions. This suggests that NONMMUG034299.1 may be co-transcribed with Cyp3a25 and that NONMMUG034299.1 transcription requires transcriptional machinery independent of Cyp3a25. The transporter Slc28a2, which is a sodium-coupled nucleoside transporter and is particularly important for the uptake of purines and may influence the pharmacokinetics of tenofovir [37], paired with the lncRNA NONMMUG024460.2, and both transcripts are transcribed from the Watson strand and were co-up-regulated in the absence of gut microbiota. Other examples of PCG paired with a lncRNA include the Phase I drug metabolizing enzymes aldo-keto reductase family 1, member C19 (Akr1c19) and Cyp27a1 (also the rate limiting step in the alternative pathway of bile acid synthesis); the Phase II drug metabolizing enzymes Aldh18a1 and sulfotransferase family, cytosolic, 1C, member 2 (Sult1c2); as well as the metal-binding protein and oxidative stress marker Mt2 (Additional file 2: Figure S3). The genomic locations of these lncRNA-PCG pairs are shown in Additional file 2: Figure S9.

#### Jejunum

**Fig. 7:**
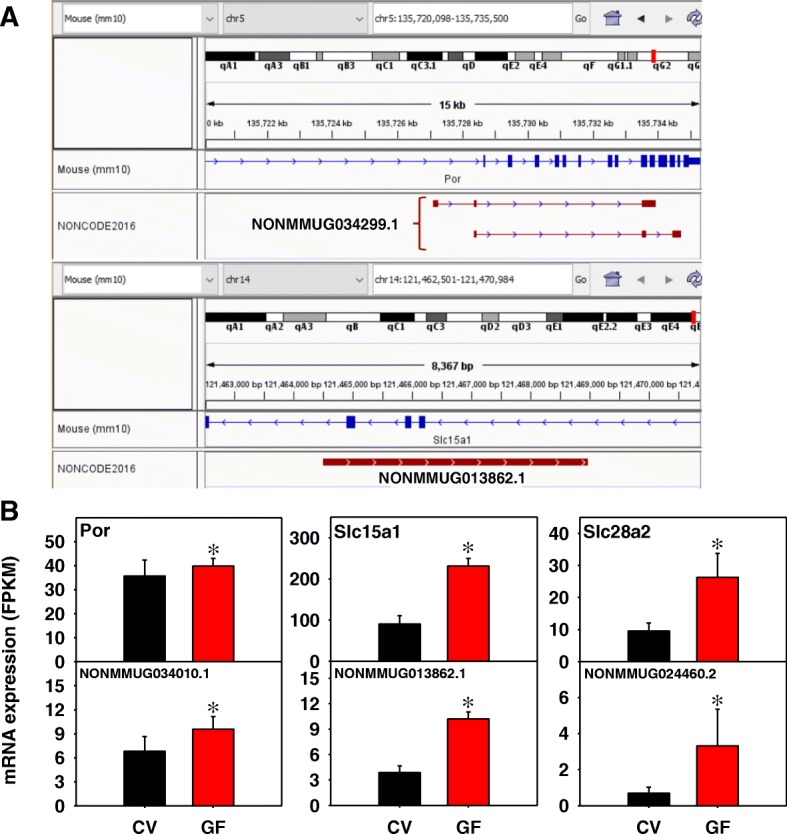
Genomic locations (**a**) and gene expression (**b**) of examples of lncRNA-PCG pairs that were differentially regulated by lack of gut microbiota in jejunum of CV and GF mice. (Note: Pathway analysis of PCGs paired with lncRNAs is shown in Additional file 2: Figure S4.) The P450 electron donor cytochrome p450 oxidoreductase (Por), the intestinal hydrogen peptide cotransporter Slc15a1, and the sodium-dependent purine transporter Slc28a2 are shown. The genomic locations for Slc5a12 and Slc28a2 are shown in Fig. 6. Expression of lncRNAs and paired PCGs were plotted using mean FPKM ± S.E., and asterisks (*) indicate statistically significant differences between enterotypes of mice (FDR-BH < 0.05)

STRING analysis of 324 PCGs paired with a lncRNA in jejunum of GF mice revealed 291 edges with a PPI enrichment *p*-value of 3.33e^− 12^ (Additional file 2: Figure S3), indicating a highly non-random set of proteins. The lncRNA NONMMUG034299.1 consists of two transcript isoforms, as shown in Fig. 7a, from the intronic region of P450 (cytochrome) oxidoreductase (Por), which is responsible for donating electrons from NADPH to P450 enzymes. Both NONMMUG034299.1 and Por are transcribed from the Watson strand and were co-up-regulated in the absence of gut microbiota (Fig. 7b). The lncRNA NONMMUG013862.1 is another intronic lncRNA transcribed from the Watson strand, and it paired with the intestinal hydrogen peptide cotransporter Slc15a1 (PepT1), and both were up-regulated by lack of gut microbiota. Similar to duodenum, the lncRNA NONMMUG024460.2 the paired sodium-coupled nucleoside transporter Slc28a2 were co-up-regulated in the absence of gut microbiota. The up-regulation of this lncRNA and Slc28a2 indicates a consistent increase in sodium and purine transport in the upper portions of the small intestine that is in part regulated by gut microbiota. Additional examples of the expression of other lncRNA-PCG pairs are shown in Additional file 2: Figure S5 and are categorized by Phase I and II drug metabolizing enzymes as well as transporters. The Phase I enzymes included bile acid-synthesizing enzymes such as Akr1b7 and Cyp27a1. The transporters included several nutrient uptake transporters, including the hydrogen peptide cotransporter Slc15a1, the creatine transporter Slc6a8, the nucleoside transporter Slc28a2, and the amino acid transporters Slc6a7, Slc7a8, Slc36a1, and Slc43a2. The genomic locations of these lncRNA-PCG pairs are shown in Additional file 2: Figure S9.

#### Ileum

**Fig. 8:**
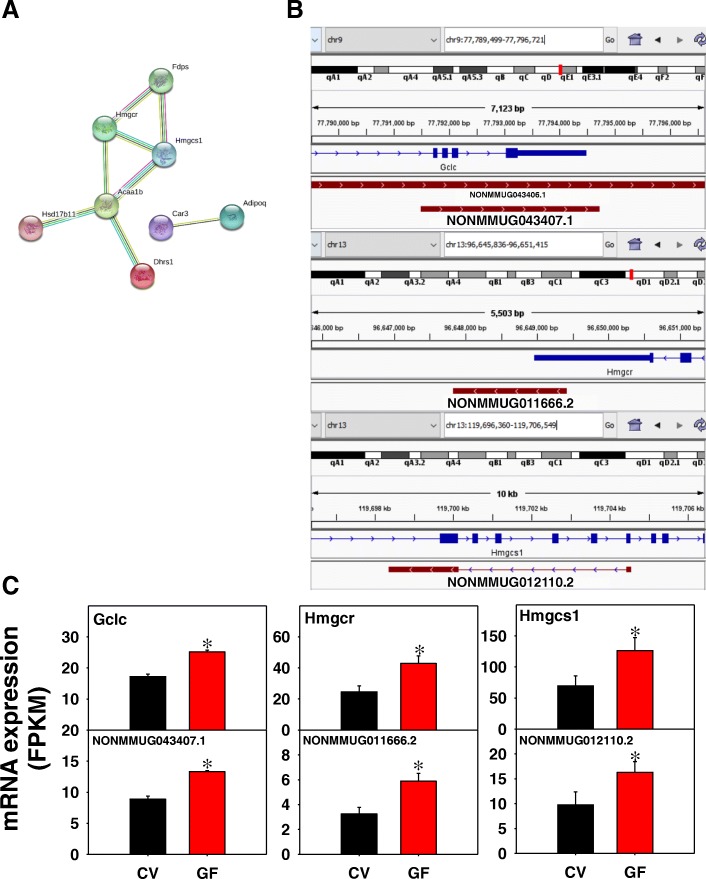
Pathway analysis (**a**), as well as genomic locations (**b**) and gene expression (C) of lncRNA-PCG pairs that were differentially regulated by lack of gut microbiota in Ileum of CV and GF mice. (**a**) The PCGs paired with lncRNAs in ileum between CV and GF mice were subjected to STRING analysis using the default settings. The connected nodes are shown. (**b**) and (**c**) The first rate-limiting enzyme of glutathione synthesis glutamate-cysteine ligase catalytic subunit (Gclc), the rate-limiting enzyme for cholesterol synthesis 3-hydroxy-3-methylglutaryl-CoA reductase (Hmgcr), and the cholesterol synthesis enzyme 3-hydroxy-3-methylglutaryl-CoA synthase 1 (Hmgcs1) are shown. Expression of lncRNAs and paired PCGs were plotted using mean FPKM ± S.E., and asterisks (*) indicate statistically significant differences between enterotypes of mice (FDR-BH < 0.05)

As shown in Fig. 8a, in ileum of GF mice 8 edges were present from 31 unique PCGs with a PPI enrichment *p*-value of 0.0002. A single enriched KEGG pathway was identified as terpenoid backbone biosynthesis, which are hydrocarbons that are precursors to steroids and strolls (Pathway ID: 00900; FDR = 0.0008). Examples of the lncRNA-PCG pairs are shown in Fig. 8b and c. The lncRNA NONMMUG043407.1 is 3223 bp in length and has a TSS in the intronic region of the first glutathione synthesis rate-limiting enzyme glutamate-cysteine ligase catalytic subunit (Gclc), ending downstream of the 3’UTR. Both NONMMUG043407.1 and Gclc were up-regulated in ileum by lack of gut microbiota, suggesting that NONMMUG043407.1 may regulate glutathione synthesis through regulating the expression of Gclc. Similarly, the lncRNA NONMMUG011666.2 is transcribed from the 3’-UTR of the cholesterol rate-limiting enzyme 3-hydroxy-3-methylglutaryl-Coenzyme A reductase (Hmgcr). Both NONMMUG011666.2 and Hmgcr are transcribed from the Crick strand and were up-regulated in the absence of gut microbiota. Overlapping transcription from the 3’-UTR suggests that Hmgcr and NONMMUG011666.2 are co-transcribed, and the local production of NONMMUG011666.2 may function as a “microRNA sponge” to prevent the microRNA-binding to the 3’-UTR of Hmgcr, and thus stabilize the mRNA and increase the protein synthesis of Hmgcr. The lncRNA NONMMUG012110.2 is an intronic lncRNA transcribed from the Crick strand in the opposite direction of 3-hydroxy-3-methylglutaryl-coenzyme A synthase 1 (Hmgcs1), which encodes the first enzyme in cholesterol biosynthesis pathway. Both NONMMUG012110.2 and Hmgcs1 were increased in the absence of gut microbiota. Additional examples of the expression of other lncRNA-PCG pairs are shown in Additional file 2: Figure S6. Phase I drug-metabolizing enzymes included two reductases Akr1c19 and Dhrs1 and the oxidizing enzyme Cyp3a25. The paired transporter Slc9a3 regulates the cellular sodium gradient. The genomic locations of these lncRNA-PCG pairs are shown in Additional file 2: Figure S9.

#### Colon

**Fig. 9:**
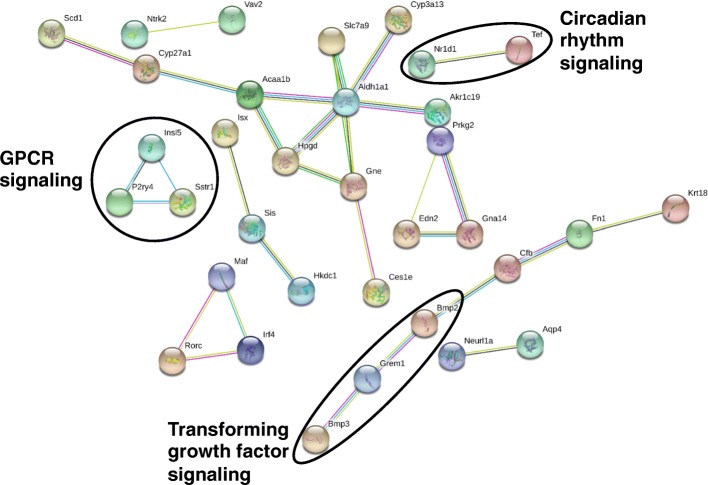
Pathway analysis of PCGs paired with lncRNAs that were differentially regulated by lack of gut microbiota in colon of CV and GF mice. The lncRNA-PCG pairs in colon between CV and GF mice were subjected to STRING analysis using the default settings. The connected nodes are shown

**Fig. 10:**
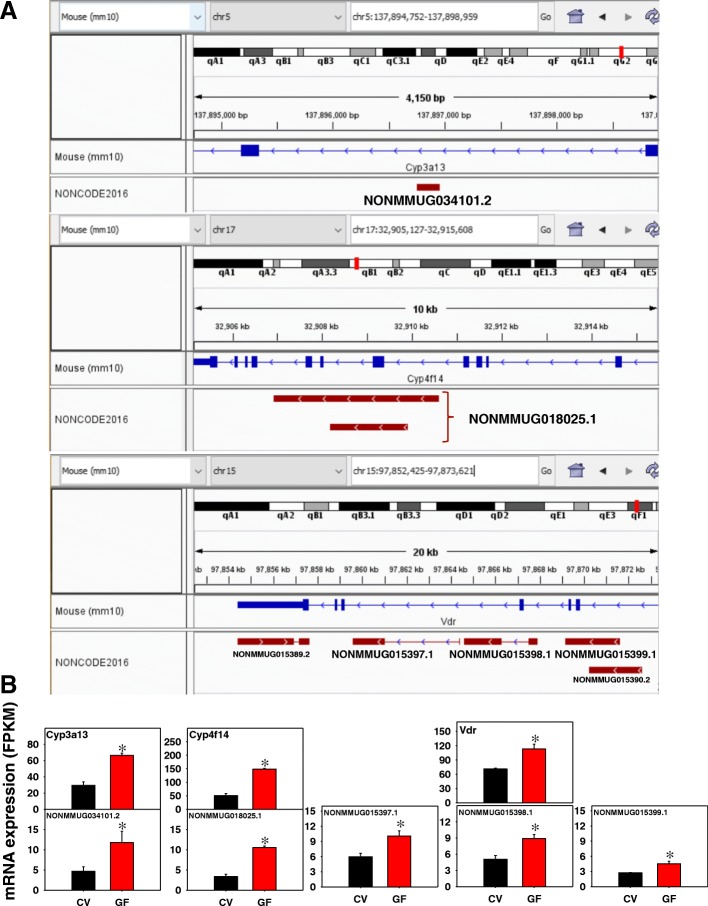
Genomic locations (**a**) and gene expression (**b**) of lncRNA-PCG pairs that were differentially regulated by lack of gut microbiota in colon of CV and GF mice. The phase-I oxidation enzyme Cyp3a13, the vitamin E hydroxylase Cyp4f14, and the vitamin D receptor (Vdr) are shown. Expression of lncRNAs and paired PCGs were plotted using mean FPKM ± S.E., and asterisks (*) indicate statistically significant differences between enterotypes of mice (FDR-BH < 0.05)

As shown in Fig. 9, in colon of GF mice, 31 edges were apparent among 111 unique PCGs with a PPI enrichment *p*-value of 0.003. Three groups of connected genes are highlighted in circles that are involved in 1) circadian rhythm signaling: the negative regulatory transcription factor nuclear receptor subfamily 1, group D, member 1 (Nr1d1) and the transcription factor thyrotroph embryonic factor (Tef); 2) G-protein-coupled receptor signaling: insulin-like 5 (Insl5), the uridine responsive receptor pyrimidinergic receptor P2Y, G-protein coupled, 4 (P2ry4), and the somatostatin-14 preferred receptor somatostatin receptor 1 (Sstr1); and 3) transforming growth factor signaling: bone morphogenetic protein 2 (Bmp2), Bmp3, and gremlin 1, DAN family BMP antagonist (Grem1). Examples of lncRNA-PCG pairs are shown in Fig. 10a and b. The lncRNA NONMMUG034101.2 on chromosome 5 is an intronic lncRNA from the drug metabolizing enzyme Cyp3a13. Both NONMMUG034101.2 and Cyp3a13 were up-regulated by the lack of gut microbiota. Another intronic lncRNA NONMMUG034101.2 has two isoforms transcribed from chromosome 17. The paired PCG is the vitamin E hydroxylase Cyp4f14, and both the lncRNA and PCG are transcribed from the Crick strand and were up-regulated in the absence of gut microbiota. The vitamin D receptor (Vdr) paired with three differentially expressed lncRNAs within the intronic region, and all three lncRNAs are transcripts are from the Crick strand. The first lncRNA NONMMUG015397.1 is from an intronic region of Vdr whereas the second lncRNA NONMMUG015398.1 spans across two adjacent introns but does not overlap with the middle exon. The third lncRNA NONMMUG015399.1 is continuously transcribed over an intron-exon region. All of the three lncRNA transcripts as well as Vdr mRNA were up-regulated in the absence of gut microbiota, and this may result from the trans-activation of one nascent transcript followed by alternative splicing at different splice sites. The expression of other lncRNA-PCG pairs is shown in Additional file 2: Figure S7 and are categorized by Phase I and drug metabolizing enzymes, transporters, and nuclear receptors. Similar to liver, the lncRNA-PCG pairs for Akr1c19 and Cyp27a1 were present in colon. Interestingly, the clock regulating nuclear receptor Nrd1d was co-expressed with NONMMUG007536.2, suggesting a co-regulatory pathway. The genomic locations of these lncRNA-PCG pairs are shown in Additional file 2: Figure S9.

#### Brown adipose tissue

**Fig. 11:**
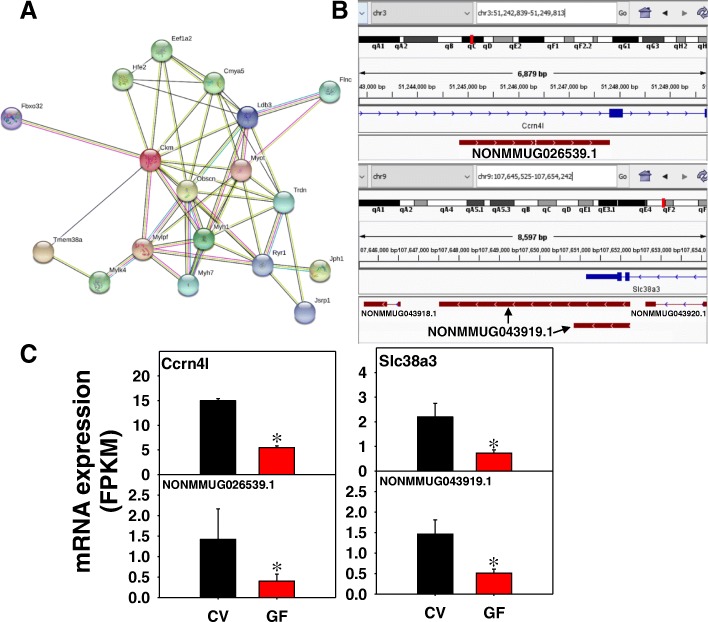
Pathway analysis (**a**), as well as genomic locations (**b**) and gene expression (**c**) of lncRNA-PCG pairs that were differentially regulated by lack of gut microbiota in BAT of CV and GF mice. (**a**) The PCGs paired with lncRNAs between CV and GF mice in BAT were subjected to STRING analysis using the default settings. The connected nodes are shown. (**b**) and (**c**) The circadian rhythm gene nocturnin (Noct/Ccrn4l) and the glutamine and sodium ion cotransporter Slc38a3 are shown. Expression of lncRNAs and paired PCGs were plotted using mean FPKM ± S.E., and asterisks (*) indicate statistically significant differences between enterotypes of mice (FDR-BH < 0.05)

As shown in Fig. 11, in BAT of GF mice, 50 edges were present from 53 unique PCGs with a PPI enrichment *p*-value less than 1e^− 16^. Examples of lncRNA-PCG pairs are shown in Fig. 11b and c. The lncRNA NONMMUG026539.1 is from the intronic region of the circadian rhythm gene nocturnin (Noct/Ccrn4l) on chromosome 3. Both NONMMUG026539.1 and Ccrn4l are transcribed from the Watson strand and were down-regulated in the absence of gut microbiota. Ccrn4l knockout mice develop lipid droplets in BAT, suggesting that down-regulation of Ccrn4l may regulate lipid metabolism, and the co-regulated NONMMUG026539.1 may also participate in this pathway [38–40]. The lncRNA NONMMUG043919.1 on chromosome 9 consists of two isoforms both transcribed from the 3’-UTR (Fig. 11b) of the sodium coupled neutral amino acid transporter Slc38a3. Both NONMMUG043919.1 and Slc38a3 are transcribed from the Crick strand and were down-regulated in the absence of gut microbiota. Additionally, the imprinted maternally expressed lncRNA H19 is down-regulated by lack of gut microbiota (Additional file 2: Figure S8).

#### White adipose tissue

**Fig. 12:**
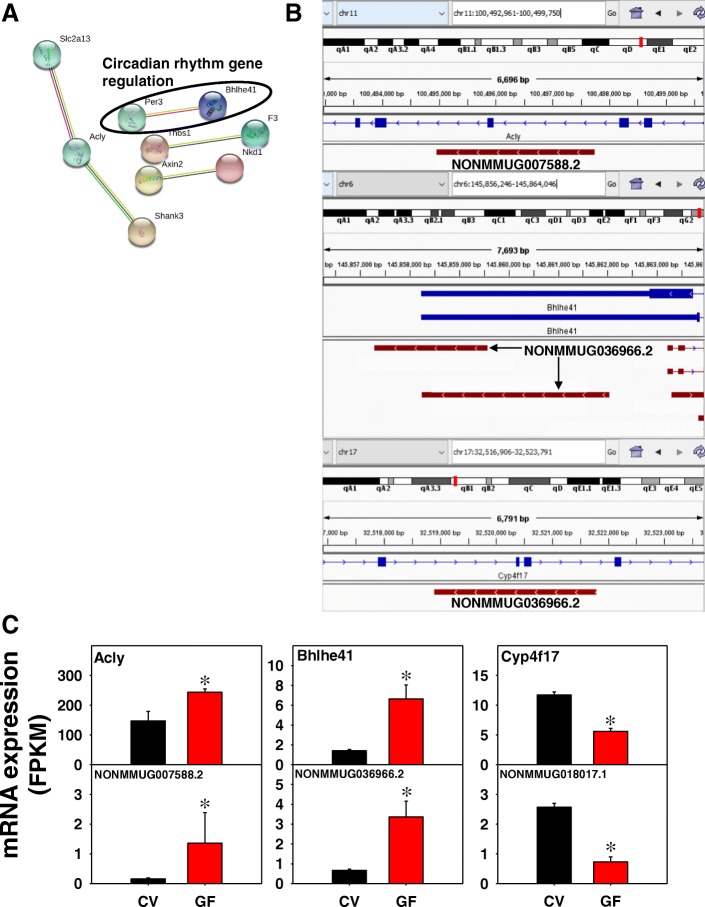
Pathway analysis (**a**), as well as genomic location (**b**) and gene expression (**c**) of lncRNA-PCG pairs that were differentially regulated by lack of gut microbiota in WAT of CV and GF mice. (**a**) The PCGs paired with lncRNAs between CV and GF mice in BAT were subjected to STRING analysis using the default settings. The connected nodes are shown. (**b**) and (**c**) The cytosolic acetyl-CoA synthesis enzyme ATP citrate lyase (Acly), the circadian rhythm regulatory gene basic helix-loop-helix family, member e41 (Bhlhe41), and the phase-I oxidation enzyme Cyp4f17 are shown. Expression of lncRNAs and paired PCGs were plotted using mean FPKM ± S.E., and asterisks (*) indicate statistically significant differences between enterotypes of mice (FDR-BH < 0.05)

As shown in Fig. 12a, in WAT of GF mice, 5 edges were apparent from 36 unique PCGs with a PPI enrichment *p*-value of 0.24, suggesting that these proteins do not have associated functions. Examples of lncRNA-PCG pairs are shown in Fig. 11b and c. The lncRNA NONMMUG007588.2 on chromosome 11 is a 2790 bp transcript from the intergenic region of the cytosolic acetyl-CoA synthesis enzyme ATP citrate lyase (Acly). Both NONMMUG007588.2 and Acyl are from the Crick strand and were co-up-regulated in the absence of gut microbiota. The lncRNA NONMMUG036966.2 consists of two transcripts from the 3’-UTR region of the circadian rhythm regulatory gene basic helix-loop-helix family, member e41 (Bhlhe41). Both NONMMUG036966.2 and Bhlhe41 are transcribed from the Crick strand and were up-regulated in the absence of gut microbiota. The lncRNA NONMMUG036966.2 is transcribed from the Crick strand of the intronic region of the oxidoreductase Cyp4f17, which is transcribed from the opposite strand. Both NONMMUG036966.2 and Cyp4f17 were down-regulated in the absence of gut microbiota.

#### Skeletal muscle

**Fig. 13:**
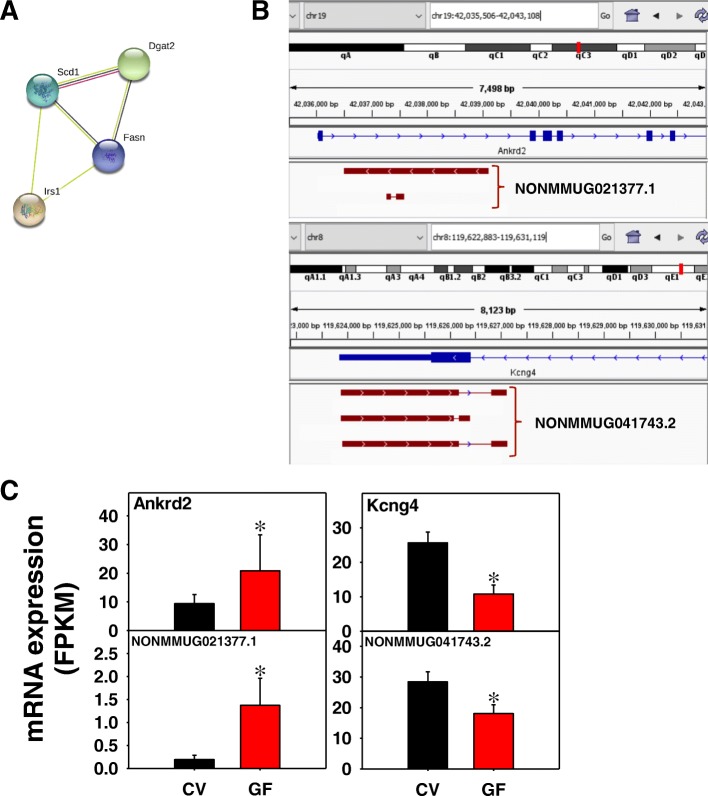
Pathway analysis (**a**), as well as genomic locations (**b**) and gene expression (**c**) of lncRNA-PCG pairs that were differentially regulated by lack of gut microbiota in skeletal muscle of CV and GF mice. (**a**) The PCGs paired with lncRNAs between CV and GF mice in skeletal muscle were subjected to STRING analysis using the default settings. The connected nodes are shown. (**b**) and (**c**) The muscle stretch response gene ankyrin repeat domain 2 (Ankrd2) and the voltage-gated potassium channel protein potassium voltage-gated channel, subfamily G, member 4 (Kcng4) are shown. Expression of lncRNAs and paired PCGs were plotted using mean FPKM ± S.E., and asterisks (*) indicate statistically significant differences between enterotypes of mice (FDR-BH < 0.05)

As shown in Fig. 13a, in muscle of GF mice, 5 edges were present from 15 unique PCGs with a PPI enrichment *p*-value of 0.0001. Enriched KEGG pathways included AMPK signaling pathway (Pathway ID: 04152; FDR = 0.0003), FoxO signaling pathway (Pathway ID: 04068; FDR = 0.012), and fatty acid metabolism (Pathway ID: 01212; FDR = 0.047). Two examples of lncRNA-PCG pairs are shown in Fig. 13b and c. The lncRNA NONMMUG021377.1 on chromosome 19 consists of two isoforms transcribed from the intronic region near the TSS of the muscle stretch response gene ankyrin repeat domain 2 (Ankrd2). NONMMUG021377.1 is transcribed from Crick strand whereas Ankrd2 is from the Watson strand. Both transcripts were up-regulated in the absence of gut microbiota. Conversely, the lncRNA NONMMUG041743.2 and the paired PCG, which is the potassium voltage-gated channel, subfamily G, member 4 (Kcng4), were down regulated in the absence of gut microbiota. NONMMUG041743.2 has three isoforms transcribed from the Watson strand near the TTS and 3’UTR of Kcng4, which is from the Crick strand, suggesting that NONMMUG041743.2 could regulate Kcng4 differently depending on the isoform.

### Predicted interactions between lncRNAs and the nascent mRNA transcripts of paired PCGs

To determine whether co-regulated lncRNA-PCG pairs potentially interact with each other, LncTar [41] was used to predict the putative interactions between lncRNAs and the nascent mRNA transcripts of paired PCGs by calculating the normalized binding free energy (ndG). Twenty-five lncRNA-PCG pairs were selected based on their importance in drug metabolism and disposition, circadian rhythm, lipid metabolism, and ion channels. Among these 25 lncRNA-PCG pairs, 12 lncRNA transcripts were predicted to bind to the nascent transcripts of the paired PCGs (Table 2) (threshold: ndG < − 0.08 based on the recommended LncTar settings). The strongest predicted binding interaction was between the lncRNA NONMMUG013862.1 and the PCG hydrogen peptide cotransporter Slc15a1, which were co-upregulated in jejunum, at − 2519 ndG. The next strongest interactions were between NONMMUG018017.1 and Cyp4f17 (co-regulated in colon and WAT), as well as between NONMMUG021377.1 and Ankrd2 (co-upregulated in muscle). These findings suggest that the paired lncRNAs may facilitate the mRNA stability. The other 13 lncRNA-PCG pairs do not appear to interact based on LncTar predictions, and it is likely that the lncRNAs share the same transcription machinery with the PCGs, but exert their functions at distal regions.

**Table 2:** Prediction of lncRNA-mRNA interactions using LncTar

LncRNA (Gene ID)	Transcript ID	PCG	ndG
NONMMUG013862.1	NONMMUT022475.1	Slc15a1	−2519.29
NONMMUG018017.1	NONMMUT029136.1	Cyp4f17	− 1570.6
NONMMUG021377.1	NONMMUT034745.1	Ankrd2	− 1507.425
NONMMUG012110.2	NONMMUT019511.2	Hmgcs1	−33.5955
NONMMUG034289.1	NONMMUT055228.1	Cyp3a11	−3.4213
NONMMUG041743.2	NONMMUT067409.2	Kcng4	−1.1499
NONMMUG041743.2	NONMMUT067411.2	Kcng4	−1.0917
NONMMUG041743.2	NONMMUT067410.2	Kcng4	−1.0911
NONMMUG030301.1	NONMMUT048798.1	Cyp4a10	−0.2888
NONMMUG034010.1	NONMMUT054821.1	Por	−0.1006
NONMMUG034101.2	NONMMUT054956.2	Cyp3a13	−0.0985
NONMMUG024462.2	NONMMUT039550.2	Slc28a2	−0.0907

### Regulation of differentially regulated PCG networks by gut microbiome in the eight target organs

To further illustrate the potential importance of lncRNAs in modifying PCG regulation by gut microbiome, we examined all differentially regulated PCGs in GF conditions in the eight target organs, regardless of whether these PCGs are paired with lncRNAs or not (Additional file 2: Figures S10-S17). In liver, the top enriched KEGG Pathways were metabolic pathways, retinol metabolism, chemical carcinogenesis, PPAR signaling pathway, and steroid hormone biosynthesis, whereas the enriched protein domains include P450s, nuclear receptors, and zinc finger proteins (Additional file 2: Figure S10). The metabolic pathways and retinol metabolism overlapped with the networks formed with PCGs that paired with lncRNAs, suggesting the involvement of lncRNAs in these processes; in contrast, chemical carcinogenesis, PPAR signaling pathway, and steroid hormone biosynthesis appeared to be independent from lncRNAs (Additional file 2: Figure S10 and Fig. 4). In addition, more P450s were enriched in networks formed by all differentially regulated PCGs in liver as compared to PCGs that paired with lncRNAs (Additional file 2: Figure S10 and Fig. 4).

In duodenum, interestingly, multiple ribosomal subunits were enriched among top networks formed by all differentially regulated PCGs (Additional file 2: Figure S11A), and this appeared to be independent from lncRNAs as the PCGs paired with lncRNAs did not form such a network (Fig. 6). The ribosomal subunits are important for protein translation process, highlighting the role of gut microbiome in protein translation in host duodenum. In addition, pancreatic secretion, chemical carcinogenesis, mineral absorption, protein digestion and absorption, and steroid hormone biosynthesis were the top enriched KEGG Pathways among all differentially regulated PCGs (Additional file 2: Figure S11B).

Jejunum had the largest numbers of both differentially regulated PCGs as well as lncRNA-paired PCGs, thus forming the largest enriched networks under both scenarios (Additional file 2: Figure S12A and Figure S4). Furthermore, the differentially regulated PCGs had greater complexity in the enriched edges in the network, indicating a lncRNA-independent effect. Virus/parasite infection, hematopoietic cell lineage, as well as protein digestion and absorption were the top most enriched KEGG pathways (Additional file 2: Figure S12B).

In ileum, the cholesterol synthesis pathway was shared between all differentially regulated PCGs (Additional file 2: Figure S13A) and lncRNA-paired PCGs (Fig. 8). However, similar to duodenum, multiple ribosomal subunits that are important for protein translation were only enriched from all differentially regulated PCGs, but not from lncRNA-paired PCGs (Additional file 2: Figure S13A and Fig. 8). In addition, many P450 isoforms as well as phase-II conjugation enzymes Gsts were enriched among all differentially regulated PCGs (Additional file 2: Figure S13A). Chemical carcinogenesis, retinol metabolism, PPAR signaling pathway, terpenoid backbone biosynthesis, and metabolic pathways were the most enriched KEGG Pathways. Overall, the ileum shared high similarities with duodenum regarding the regulation of PCGs by the absence of gut microbiome.

In colon, circadian rhythm pathway was shared between all differentially regulated PCGs and lncRNA-paired PCGs (Additional file 2: Figure S14 and Fig. 9), whereas protein digestion and absorption, metabolic pathways, fat digestion and absorption, and pancreatic secretion were enriched for all differentially regulated PCGs (Additional file 2: Figure S14), but G-protein-coupled receptor signaling and transforming growth factor signaling were enriched for lncRNA-paired PCGs (Fig. 9).

In BAT, multiple ribosomal subunits were also enriched among differentially regulated PCGs, and interesting the muscle-related pathways appeared to be regulated in this tissue by the lack of gut microbiome (Additional file 2: Figure S15A and B). In contrast, the lncRNA-paired PCGs appeared to be more involved in circadian rhythm, lipid metabolism, and amino acid transport (Fig. 11b).

In WAT, circadian rhythm pathway was shared between all differentially regulated PCGs and lncRNA-paired PCGs (Additional file 2: Figure S16 and Fig. 12). In addition, leukocyte transendothelial migration, metabolism of xenobiotics by cytochrome P450s, microbial metabolism in diverse environment were the top enriched KEGG Pathways from all differentially regulated PCGs.

In skeletal muscle, AMPK signaling and FoxO signaling pathway were shared between all differentially regulated PCGs and lncRNA-paired PCGs (Additional file 2: Figure S17 and Fig. 13), whereas circadian rhythm, metabolism of xenobiotics by cytochrome P450s and p53 signaling pathway were enriched from all differentially regulated PCGs.

To confirm RNA-Seq results, selected lncRNAs in various organs were validated by RT-qPCR as shown in (Additional file 2: Figure S18) (Note: the liver results were compared with control mice from a previously published study from this laboratory) [42]. The RT-qPCR results were consistent with the RNA-Seq data.

In summary, the lncRNA-paired PCGs share overlapping but not identical signaling pathways as compared to all differentially regulated PCGs by the absence of gut microbiome, indicating that lncRNAs may regulate a subset of the PCGs within the same neighborhood, but are not involved in certain other biological processes.

## Discussion

Gut microbiota has been shown to modulate the expression of host PCGs in liver, duodenum, jejunum, ilium, and colon [27–31, 43]. In liver of adult GF mice, the expression of the drug-metabolizing enzyme Cyp3a11 gene is lower than that of CV mice [30]. The gut microbiota-mediated modification of hepatic gene expression is regulated by remote-sensing mechanisms, through microbial metabolites that may act as activators of host transcription factors [28]. The xenobiotic-sensing nuclear receptor pregnane X receptor (PXR) can be activated by the secondary bile acid lithocholic acid, which is a product of microbial metabolism of host primary bile acids [44] and regulates Cyp3a in mouse liver [27]. Additionally, the absence of bacteria and their metabolites has been shown to modulate several metabolic processes in peripheral tissues. ^1^H NMR metabolic profiles of BAT in CV and GF mice of both genders showed that lack of gut microbiota eliminated sexual dimorphisms in GF mice and decreased lactate levels while increasing (D)-3-hydroxybutyrate, suggesting that gut microbiota may stimulate lipolysis and inhibit lipogenesis in BAT [45]. In skeletal muscle and BAT, it has been suggested that propionic acid, a short chain fatty acid (SCFA) produced by gut microbiota, acts as a mediator to increase the expression of uncoupling protein (UCP)-1 for thermogenesis and energy expenditure [46]. Mice with a depleted gut microbiota exposed to the gut microbiota of mice fasted every other day increased total energy expenditure and the expression of UCP to induce the beiging of WAT; increased circulatory levels of the SCFA acetate and the fermentation product lactate are the suggested underlying mechanism [47]. Because lncRNAs have been increasingly recognized as critical regulators for various metabolic processes [48–51], it is possible that lncRNAs may at least partially contribute to the regulation of PCGs and intermediary metabolism pathways by gut microbiota. Prior to this study, using an Affymetrix mouse exon microarray dataset, Liang et al., (2015) identified lncRNA expression profiles in intestinal epithelial cells isolated from CV and GF mice, as well as GF mice recolonized with *Escherichia coli*, suggested that some lncRNAs may be microbe-dependent [52]. In an in vitro experiment of endothelial cells, the bacterial membrane component lipopolysaccharide differentially regulated the expression of over 19,000 lncRNA transcripts [53]. In the present study, we showed that the presence of gut microbiota is necessary in the constitutive expression of lncRNAs in multiple tissues of mice, including liver, duodenum, jejunum, ileum, colon, BAT, WAT, and muscle. These results have demonstrated for the first time that lncRNAs are regulated by microbial metabolites both proximal (intestine sections) and distal (liver, fat, and muscle) to the gut microbiota.

Interestingly, this study showed that within the enterohepatic tissues (liver and intestinal sections) of CV mice, the majority of the expressed lncRNAs were co-expressed across these tissues (5359) (Fig. 1b). Similarly, in the peripheral metabolic organs (BAT, WAT, and muscle), the majority of the expressed lncRNAs were also co-expressed (4423). Similar co-expression patterns were also observed in rainbow trout with more lncRNAs (3269) being co-expressed in all tested tissues than tissue-specific lncRNAs (2935) [54] and across 18 tissues in a bovine model [55]. However, this study showed that in the absence of gut microbiota, differentially regulated lncRNAs is highly tissue-specific because only three out of 1354 lncRNAs were commonly regulated by lack of gut microbiota in enteroheptic organs and only one out of 310 lncRNAs was commonly regulated by lack of gut microbiota in peripheral metabolic tissues (Fig. 2b). This suggests that lncRNAs regulated by gut microbiota-mediated signaling have distinct roles in defining tissue specific differentiation and functions.

The gut microbiota-mediated differentially expressed PCGs that paired with lncRNAs were clustered into distinct functional networks (Figs. 1a, 4a, 6, 8b, 9a, 11, 12a and Additional file 2: Figure S4), suggesting that lncRNAs may modulate these pathways in the absence of gut microbiota. Indeed, it has been demonstrated in human naïve central memory and effector memory CD4^+^ T cells that lineage-specific lncRNAs were co-expressed with lineage-specific PCGs [56], and lncRNA expression profiles in intestinal tissues can discriminate between different types of bacteria [52]. Both reports and the present study have suggested that lncRNAs contribute to the regulation of the lineage PCGs and cellular phenotypes.

Among the seven tissues examined, we found that the majority of differentially regulated lncRNAs by lack of gut microbiota (Fig. 3) were transcribed from the introns of PCGs. Previously, the majority of transcripts mapped to intronic regions of the genome were dismissed from functional characterization as they were thought to be mRNA precursors or transcriptional noise. A comprehensive analysis of transcriptional output in humans and mice revealed that there are at least 78,147 and 39,660 intronic transcripts, respectively, with some evidence for conservation between species [57]. The function of many lncRNAs, in particular intronic lncRNAs, is hypothesized to act though *cis* mechanisms to modulate the transcription of PCGs. It is increasingly recognized that intronic lncRNAs may regulate the RNA processing pathway of PCGs, including transcription splicing, and translation [58], and it has been suggested that the interaction of lncRNAs within introns of PCGs may have a synergistic effect for “fine-tuning” gene expression patterns [59]. Plausibly, these lncRNAs regulate the post-transcriptional splicing of nascent PCG transcripts, and compared to lncRNAs mapped to other genomic regions, these intronic lncRNAs are more readily regulated by gut microbiota.

The second most prevalent genomic location of differentially regulated lncRNAs by lack of gut microbiota was the intergenic region, which do not have an immediate PCG to regulate in *cis*. The intergenic lncRNA HOTAIR was the first to be characterized to act in *trans* and is required to maintain transcriptional silencing of the *HOXD* locus on chromosome 2, but is transcribed antisense to the *HOXC* locus on chromosome 12 [9]. Previous efforts in identifying functional intergenic lncRNAs have focused on loci with permissive epigenetic marks with a major emphasis on short regions with histone H3 lysine 4 tri-methylation (H3K4Me3) corresponding to gene promoters followed by longer regions with histone H3 lysine 36 tri-methylation (H3K36Me3) corresponding to the transcribed region [60–63]. Using permissive epigenetic marks as a method to identify nascent lncRNAs has suggested that there are about 4500 evolutionarily conserved intergenic transcripts in humans and that up to 38% form chromatin-modifying complexes, organizing epigenetic enzymes spatially for gene regulation [63]. Lack of gut-microbiota dys-regulated many intergenic lncRNAs, suggesting that differences in gene expression may be due to microbiota-dependent epigenetic gene regulation. Indeed, it has been suggested that gut microbiota may regulate epigenetic control of host gene expression [64–66]. The crosstalk between gut microbiota and the host epigenome has recently been reviewed by Qin and Wade [67]. LncRNAs transcribed from other regions, such as the 5′- and 3’-UTR been suggested to alter post transcriptional processes through alternative splicing, acting as decoys (competing endogenous RNA) for miRNA inhibition and improving translational stability [68–72]. Using the lncRNA transcription loci relative to PCGs as a predictor of function, the data suggest lncRNAs regulated by lack of gut microbiota may have diverse roles in the regulation of epigenetic enzymes and the stability of mRNAs during post-transcriptional processing and translation.

The expression patterns between lncRNA-PCG pairs has been demonstrated to be highly correlated [73]. We found that nearly all differentially expressed lncRNA-PCG pairs to be co-regulated by the lack of gut microbiota, suggesting that lncRNAs and neighboring PCGs may share common promoters and/or enhancers for transcription. It is also possible that a lncRNA-PCG pair may be first transcribed as one nascent transcript and then cleaved into the lncRNA and PCG mature transcripts. The present study showed that jejunum had over two-fold more differentially expressed lncRNAs than any other tissue (Fig. 2a) and the most lncRNA-PCG pairs (Additional file 1: Table S2). Jejunum has is a primary site for the absorption of nutrients, such as the passive transport of fructose and the active transport of amino acids, peptides, vitamins, and glucose. Several lncRNAs in jejunum paired with solute transporters including the hydrogen peptide cotransporter Slc15a1, the creatine transporter Slc6a8, the nucleoside transporter Slc28a2, and the amino acid transporters Slc6a7, Slc7a8, Slc25a22, Slc36a1, and Slc43a2. Interestingly, Slc28a2 is a sodium-dependent transporter and Slc36a1 is a proton dependent transporter, indicating that lncRNAs may help regulate the pH of the lumen. Additionally, the di- and tri-uptake transporter Slc15a1 was strongly predicted to interact with the paired lncRNA NONMMUG013862.1. The co-expression of these PCGs with lncRNAs suggests that gut microbiota may regulate the absorption of nutrients through lncRNAs as well as the pH of the intestines.

Recently, lncRNAs were identified to have unique promoter regions with transcription factor binding motifs distinct from PCGs [74]. Many of the identified transcription factors were also known to be regulated by the lncRNAs that contain the distinct binding motif in their promoter region, which is suggestive of a self-regulatory feedback loop [74]. Many enhancer regions are also transcribed into lncRNAs (referred to as enhancer RNAs, eRNAs) and are correlated with the activity of functional enhancers [75]. It has been suggested that eRNAs may serve as important regulators of enhancer activation through spatial enhancer-promoter looping and temporal trafficking of transcriptional machinery [75–77]. Indeed, analysis of the sponge (*Amphimedonqueenslandica*) suggests that *cis*-regulation by non-coding elements in introns of nearby functionally unrelated genes constrains the evolution of the surrounding genes [78], and experimental evidence in vertebrates supports this hypothesis [79, 80].

It should be noted that in the present study, we used poly-A tail selection method to enrich mRNAs and poly-A tailed lncRNAs during library preparation. This potentially introduces sampling bias because not all lncRNAs are poly-A tailed. An analysis of 27 human RNA-Seq libraries that used RiboMinus depletion of rRNA (not poly-A dependent) captured 24.1% of transcripts. However, only 1.7% of the expressed sequences were uniquely expressed to these libraries compared to 36 datasets that used polyA selection [81]. In the human transcriptome, at least 39% of lncRNAs in the human transcriptome contain one of the six common poly-adenylation motifs, compared to 51% of coding transcripts [4]. The surprisingly similar yield between poly-A selection versus ribosomal depletion may be due to the tissue specificity of lncRNAs as well as their low expression. It has been shown that the non-polyadenylated lncRNAs are generally expressed at lower levels than the non-polyadenylated mRNAs and are prevalent in the nucleus, suggesting that they may be involved in the transcriptional regulation of target genes [82, 83]. At this time, the ratio between polyadenylated lncRNAs versus non-polyadenylated lncRNAs in liver is not known and most of the well characterized lncRNAs are produced using the same machinery as the PCGs (i.e. transcribed by RNA Pol-II, polyadenylated, and spliced) [84]. Because the present study was based on our previously published RNA-Seq dataset using poly-A selection strategy (NCBI GEO database GSE10474) [29, 42, 81), we were not able to examine the non-poly-A tailed lncRNAs within this scope. Future studies using whole transcriptome analysis could use ribosomal depletion as an alternative library construction option (although the trade-off will be lower signal per transcript at the same read depth).

One question that arises is whether the paired lncRNAs are true signals versus ontly transcriptional noise of PCGs. Interestingly, the lncRNAs that were paired with PCGs shown in Figs. 5, 6, 7, 8, 9, 10, 11, 12 all overlapped with the PCGs. However, there were many PCG-paired lncRNAs that were located upstream or downstream of the PCGs (Additional file 1: Table S2) in all eight organs investigated. Secondly, the present study utilized poly-A tail selection strategy to remove the ribosomal RNA from the total RNA species during library construction of RNA-Seq, eliminating the possibility that the lncRNAs are transcriptional noise of PCGs. Therefore, only the mature lncRNAs as well as the mature mRNAs are captured in the final output, and the detected signal should be true lncRNA transcripts rather than transcriptional noise of PCGs.

The present study showed that approximately 10% of lncRNAs were distributed within exonic regions of PCGs (Fig. 3). Functionally, the lncRNAs that overlap with PCGs may affect the regulation of PCGs at multiple levels including transcription, mRNA splicing and stability, as well as cellular transport and protein translation. Regarding read assignments of the overlapping exons, the multi-mapping reads are typically accounted for by using a statistical model that probabilistically assigns such reads while inferring maximum likelihood estimates of transcript abundance. In the mouse genome, the majority of the exons are between 50 and 150 bp in length, and in the present study, the sequencing strategy was 50 bp paired end with an average insert size of approximately 160 bp. Therefore, we expect that a substantial amount of reads span across the junctions of the two adjacent exons of the mature transcripts derived from both lncRNAs and PCGs. The mapping strategy using HISAT starts by trying to find candidate locations of part of each read using the global Ferragina-Manzini (FM) index. From there, it selects one of the local indexes (approximately 48,000 in total) for each candidate location, and then uses this to align the remaining portion of each read. The forward and reserve reads were separately aligned, and then combined from both ends. Therefore, using the current mapping method, we expect that lncRNAs that have overlapping exons but do not have completely identical two adjacent exons to PCGs will be differentiated from the corresponding PCGs.

## Conclusions

Taken together, the present study is among the first to demonstrate that lack of gut microbiota differentially regulates the expression of lncRNAs not only within intestine, but also in other important metabolic organs (liver, fat, and muscle). Interestingly, the majority of gut microbiota-regulated lncRNAs were in jejunum, which is the primary section of intestine responsible for absorption. Nearly all lncRNA-PCG pairs were co-regulated (i.e. both either up- or down-regulated) in the absence of gut microbiota. STRING analysis showed that differentially expressed PCGs in proximity to lncRNAs form tissue-specific networks, suggesting that lncRNAs may interact with gut microbiota locally or remotely to regulate tissue-specific functions.

## Methods

### Animals and procedures

**Fig. 14:**
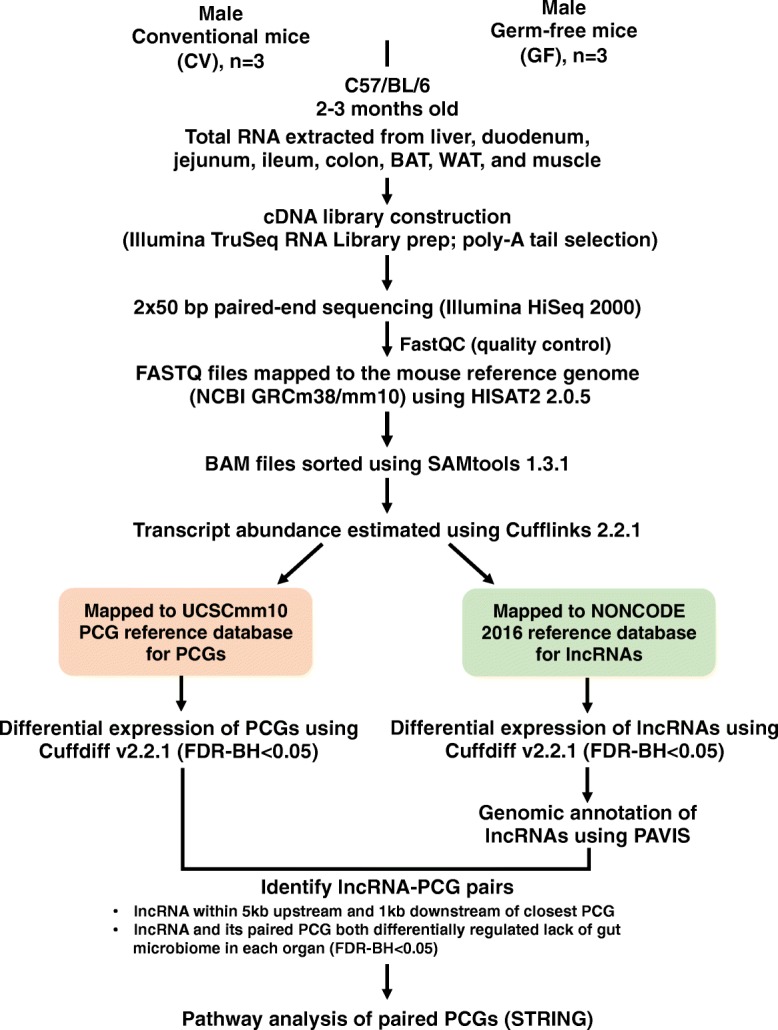
Diagram illustrating the experimental design and workflow for RNA-seq data analysis. Briefly, tissues from 2 to 3-month-old C57BL/6 conventional (CV) and germ-free (GF) mice were harvested as described previously [29]. Total RNAs were extracted from each organ (*n* = 3 per enterotype per organ). The cDNA libraries were prepared using the poly-A tail selection method and were sequenced using an Illumina HiSeq2000 sequencer (2 × 50 bp paired-end). Fastq files were quality-checked with FastQC and mapped to the mouse reference genome (mm10) using HISAT. The sequence alignment mapping (SAM) files were converted to binary alignment mapping (BAM) files and sorted using SAMtools. The sorted BAM files were subjected to Cufflinks to determine the transcript abundance. Specifically, the transcript abundance of lncRNAs and PCGs was estimated using the mouse NONCODE 2016 lncRNA and UCSC mm10 PCG reference gene transfer format (GTF) files, respectively. The differentially expressed lncRNAs and PCGs were determined by Cuffdiff between CV and GF mice for each organ (FDR-BH < 0.05). The genomic annotation of differentially expressed lncRNAs and their closest PCGs were annotated using PAVIS. A lncRNA is considered paired with a proximal PCG if 1) the lncRNA overlaps with the coding region or is within 5 kb upstream of TSS or 1 kb downstream of TTS of any PCG and 2) both the lncRNA and the proximal PCG were differentially expressed between CV and GF mice (average FPKM > 1 in either CV or GF mice and FDR-BH < 0.05). The PCGs paired with lncRNAs were subjected to pathway analysis using STRING

As previously described [42, 81], all mice used in this study were male C57BL/6 mice at 2–3 months of age (*n* = 3 per group). Mice were housed with a 14-h light/10-h dark cycle in a temperature and humidity controlled environment in an Association for Assessment and Accreditation of Laboratory Animal Care International–accredited facility at the University of Kansas Medical Center (KUMC). All CV mice were purchased from The Jackson Laboratory (Bar Harbor, ME). The initial breeding colony of C57BL/6 mice was established with GF mice purchased from the National Gnotobiotic Rodent Resource Center (University of North Carolina at Chapel Hill). All mice had *ad libitium* access to autoclaved rodent chow (catalog #5 K67; LabDiet, St. Louis, MO) and autoclaved water. Mice were euthanized in a CO2 chamber (at a flow rate 20% of chamber volume for 5 min) followed by opening the abdominal cavity. As described in Fig. 14, tissues were harvested between 9:00 AM and noon. The following tissues were collected: liver, duodenum, jejunum, ileum, large intestine, BAT, WAT, and skeletal muscle. Intestinal contents were flushed with ice-cold phosphate-buffered saline. Tissues were immediately frozen in liquid nitrogen and stored at − 80 °C before further analysis.

### Total RNA isolation, DNA library construction, and RNA-sequencing

Total RNA was isolated from tissues using RNA-Bee reagent according to the manufacturer’s protocol. RNA concentrations were quantified using a NanoDrop 1000 Spectrophotometer (Thermo Fisher Scientific, Waltham, MA). Integrity of total RNA was confirmed using an Agilent 2100 Bioanalyzer (Agilent Technologies Inc., Santa Clara, CA), and samples with RNA integrity values of about 7.0 were used for sequencing. The complementary DNA (cDNA) libraries were constructed from total RNA samples using a TruSeq RNA Sample Prep Kit with poly-A tail selection (Illumina, San Diego, CA). Sequencing was performed on an Illumina HiSeq2000 sequencer using a 50 bp paired-end multiplexing strategy at the Kansas University Medical Center Genome Sequencing Facility. These procedures were described in previous publications [29, 43, 85].

### RNA-sequencing data analysis

The raw RNA-Seq data was deposited in Gene Expression Onmibus (GEO) database (accession numbers: GSE79848 and GSE102867). As shown in Fig. 14, FASTQ files containing paired-end sequence reads were mapped to the mouse reference genome (GRCm38/mm10) using HISAT2 (Hierarchical Indexing for Spliced Alignment of Transcripts) (version 2.0.5) [86]. The output SAM (sequencing alignment/map) files were converted to BAM (binary alignment/map) files and sorted using SAMtools (version 1.3.1) [63]. The transcript abundance for lncRNAs and PCGs was estimated by Cufflinks (version 2.2.1) using the NONCODE 2016 lncRNA and UCSC mm10 PCG references databases, respectively. The mRNA abundance was expressed as fragments per kilobase of exon per million reads mapped (FPKM). LncRNAs and PCGs with an average FPKM above 1 in either enterotype were considered expressed for each organ. Differential analysis between CV and GF mice was performed using Cuffdiff, and transcripts with a Benjamini-Hochberg adjusted false discovery rate (FDR-BH) < 0.05 were considered differentially regulated by lack of gut microbiome. Data were expressed as mean FPKM ± S.E., and asterisks (*) represent significant differences between CV and GF mice. Venn diagrams of lncRNAs (Figs. 1 and 14) and two-way hierarchical clustering dendrograms (Ward’s minimum variance method, distance scale; Additional file 2: Figure S1) were generated using JMP Genomics Version 8 (SAS Institute, Cary, NC).

### Genomic annotation of lncRNAs and proximal lncRNA-PCG pair identification

To annotate and visualize the genomic location of lncRNAs relative to the closest PCGs, the web-based tool peak annotation and visualization (PAVIS, https://manticore.niehs.nih.gov/pavis2/) was used to identify lncRNAs proximal to PCGs, including 5 kb upstream of the transcription start site (TSS), intronic, exonic, 5′-untranslated region (UTR), 3’-UTR, and up to 1 kb downstream of the transcriptional termination site (TTS). A lncRNA and PCG are considered paired in a certain tissue if 1) the lncRNA overlaps with or is within 5 kb upstream of TSS or 1 kb downstream of TTS of any PCG and 2) both the lncRNA and the proximal PCG were differentially expressed between CV and GF mice (FPKM > 1 in CV or GF mice and FDR-BH < 0.05). Gene structure and relative genomic location of the lncRNA-PCG pairs were visualized using Integrated Genome Viewer (Broad Institute, Cambridge, MA).

### Pathway analysis of differentially expressed lncRNA-PCG pairs

For each organ, the differentially expressed PCGs that paired with a differentially expressed lncRNA were submitted for STRING (Search Tool for the Retrieval of Interacting Genes/Proteins) analysis (version 10.5, https://string-db.org/) [36].

### LncTar

Mouse lncRNA sequences were retrieved from the NONCODE 2016 database (http://www.noncode.org/download.php) and mouse protein-coding transcript sequences were retrieved from Ensembl Biomart. LncTar Version 1.0, a command line tool for predicting lncRNA-RNA interactions, was used to generate a tab-delimited file of lncRNA and PCG pairs based on predicted free-energy associations. Specifically, we predicted the interactions between lncRNA and the paired PCG nascent transcript. A threshold of − 0.08 ndG was set because it is the lowest suggested threshold and could detect all possible lncRNA-mRNA interactions.

## Additional files

Additional file 1: **Table S1.** Mapping statistics of RNA-Seq data in various organs from CV and GF mice. Sample IDs, total reads sequenced, total reads mapped, and percentage of reads mapped to the mouse genome are shown. **Table S2.** lncRNA-PCG pairs in each organ investigated. The gene IDs, chromosome coordniates, gene expression in each individual sample per organ, log2 fold change, as well as FDR-BH (q value) for both lncRNAs and the paired PCGs are shown. **Table S3.** Regulation of intergenic lncRNAs in liver by the absence of gut mirobiome. **Table S4.** Regulation of intergenic lncRNAs in duodenum by the absence of gut mirobiome. **Table S5.** Regulation of intergenic lncRNAs in jejunum by the absence of gut mirobiome. **Table S6.** Regulation of intergenic lncRNAs in illeum by the absence of gut mirobiome. **Table S7.** Regulation of intergenic lncRNAs in colon by the absence of gut mirobiome. **Table S8.** Regulation of intergenic lncRNAs in BAT by the absence of gut mirobiome. **Table S9.** Regulation of intergenic lncRNAs in WAT by the absence of gut mirobiome. **Table S10.** Regulation of intergenic lncRNAs in skeletal muscle by the absence of gut mirobiome. (XLSX 279 kb)

Additional file 2: **Figure S1.** Two-way hierarchical clustering dendrograms of differentially expressed lncRNAs by lack of gut microbiota. **Figure S2-S8.** Other examples of the expression of lncRNA-PCG pairs that were differentially regulated by lack of gut microbiota in liver (Figure S2), duodenum (Figure S3), jejunum (Figure S4-5), ileum (Figure S6), colon (Figure S7), and BAT (Figure S8). **Figure S9.** Genomic locations of lncRNA-PCG pairs described in Additional file 2: Figures S2-S3 and S5-S8.** Figure S10-17.** All differentially regulated PCG networks (STRING analysis), enriched Biological Process (GO), Molecular Function (GO), Cellular Component (GO), and KEGG Pathways in liver (Figure S10), duodenum (Figure S11), jejunum (Figure S12), ileum (Figure S13), colon (Figure S14), BAT (Figure S15), WAT (Figure S16), and skeletal muscle (Figure S17). **Figure S18.** RT-qPCR validation of selected lncRNAs from the RNA-Seq data. (PDF 143690 kb)

## Abbreviations

Acly: ATP citrate lyase
AHR2: Aryl hydrocarbon receptor 2
Akr1c19: Aldo-keto reductase family 1, member C19
Aldh: Aldehyde dehydrogenase
Ankrd2: Ankyrin repeat domain 2
Ar: Androgen receptor
BAM: Binary alignment/map
BAT: Brown adipose tissue
Bhlhe41: Basic helix-loop-helix family, member e41
Bmp2: Bone morphogenetic protein 2
cDNA: Complementary DNA
CV: Conventional
Cyp: Cytochrome P450
eRNAs: Enhancer RNAs
FDR-BH: Benjamini-Hochberg adjusted false discovery rate
Fmo5: Flavin containing monooxygenase 5
FPKM: Fragments per kilobase of exon per million reads mapped
Gclc: Glutamate-cysteine ligase catalytic subunit
GEO: Gene Expression Onmibus
GF: Germ-free
Grem1: Gremlin 1, DAN family BMP antagonist
group D: Member 1
Gss: Glutathione synthetase
GTF: Gene transfer format
H3K27Me3: Tri-methylation of histone H3 lysine 27
H3K36Me3: Histone H3 lysine 36 tri-methylation
H3K4Me3: Histone H3 lysine 4 tri-methylation
Hmgcr: 3-hydroxy-3-methylglutaryl-CoA reductase
Hmgcs1: 3-hydroxy-3-methylglutaryl-CoA synthase 1
HOX: Homeobox
Hsd17b11: Hydroxysteroid 17a dehydrogenase
Insl5: Insulin-like 5
Kcng4: Potassium voltage-gated channel, subfamily G, member 4
KUMC: University of Kansas Medical Center
lncRNAs: Long non-coding RNA
MLL: Mixed-lineage leukemia
Mt2: Metallothionein 2
ncRNA: Non-coding RNA
ndG: Normalized binding free energy
NIH: National Institutes of Health
Noct/Ccrn4l: nocturnin
Nr1d1: Nuclear receptor subfamily 1
P2ry4: Receptor pyrimidinergic receptor P2Y, G-protein coupled, 4
PAVIS: Peak annotation and visualization
PCG: Protein-coding gene
Phldb1: Pleckstrin homology like domain, family B, member 1
Por: p450 (cytochrome) oxidoreductase
PPI: Protein-protein interaction
PRC2: Polycomb Repressive Complex 2
PXR: Pregnane X receptor
SAM: Sequencing alignment/map
SCFA: Short chain fatty acid
Slc: Solute carrier
sox: Sex-determining region Y-box
Sstr1: Somatostatin receptor 1
STRING: Search Tool for the Retrieval of Interacting Genes/Proteins
Sul1c2: Sulfotransferase family, cytosolic, 1C, member 2
Tef: Thyrotroph embryonic factor
TSS: Transcription start site
TTS: Transcriptional termination site
UCP: Uncoupling protein
UTR: Untranslated region
Vdr: Vitamin D receptor
WAT: White adipose tissue
WDR: WD repeat domain
