# Supplementary material for: Coordinate regulation of long non-coding RNAs and protein-coding genes in germ-free mice
Source: BMC Genomics. 2018 Nov 21;19:834. doi: 10.1186/s12864-018-5235-3 (PMC6249886; doi:10.1186/s12864-018-5235-3)

Supplementary Figure 1

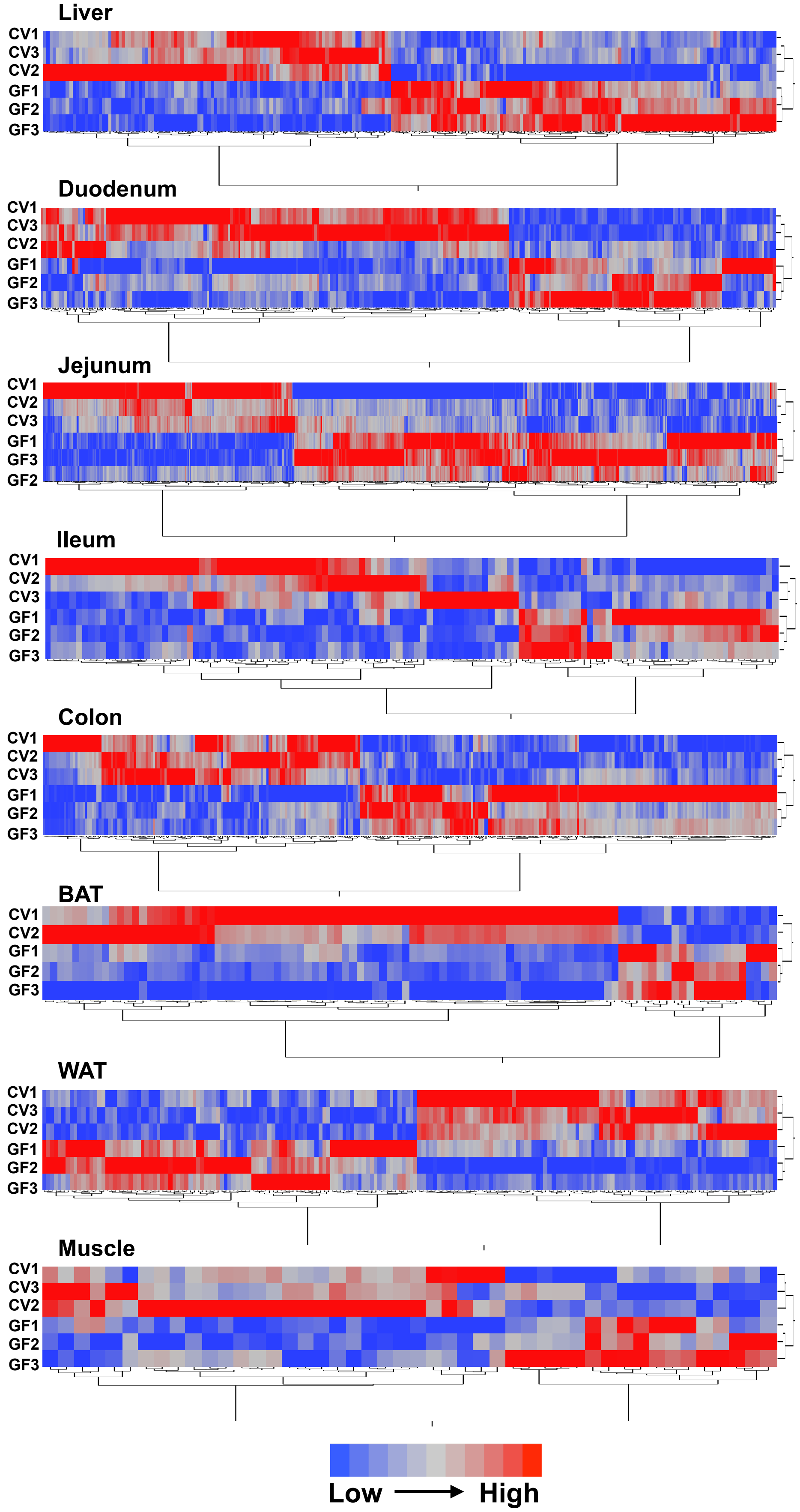

Supplementary Figure 2 Liver

A Cholesterol biosynthesis

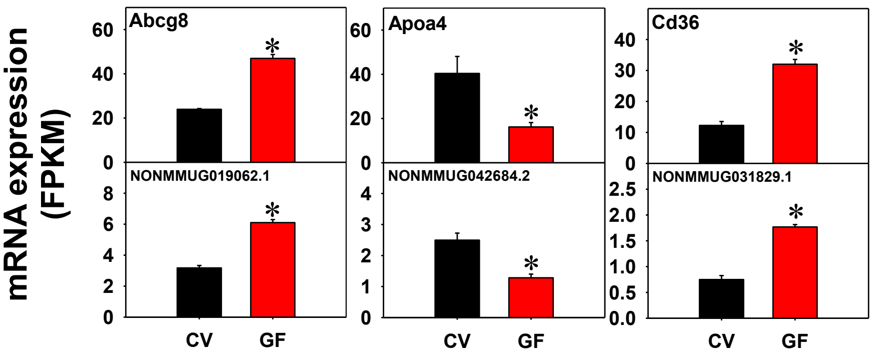

B Drug metabolism and oxidative stress

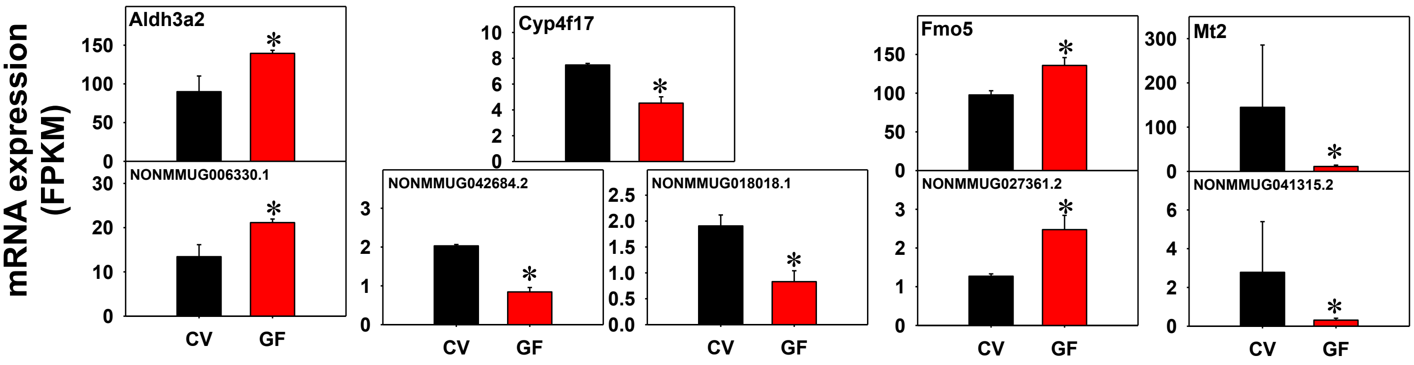

C Nucleoside transport

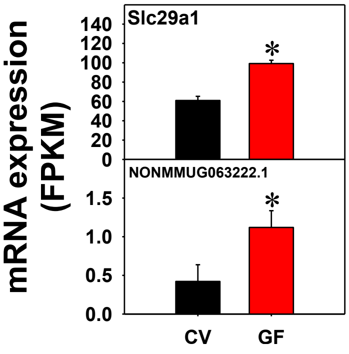

Supplementary Figure 3 Duodenum

A Drug metabolizing enzymes: Phase I

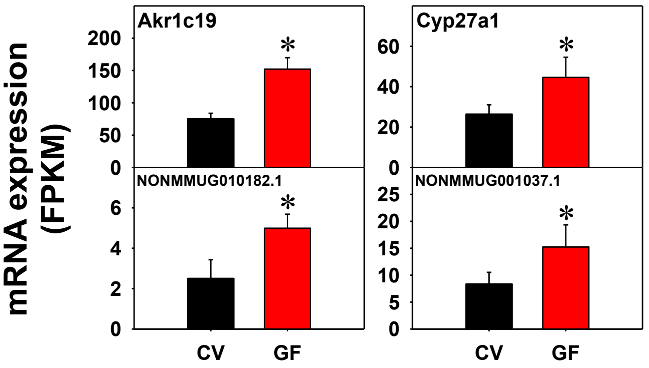

B Drug metabolizing enzymes: Phase II

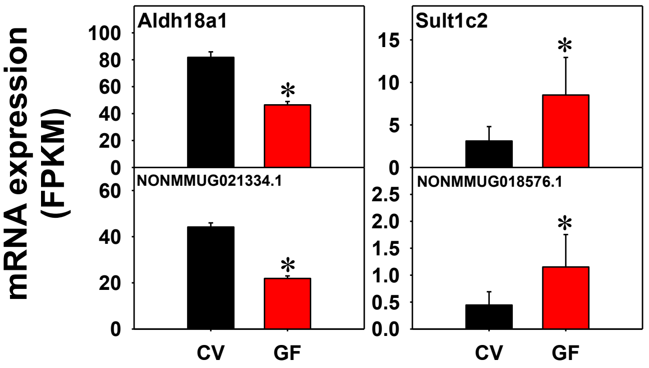

C Oxidative stress

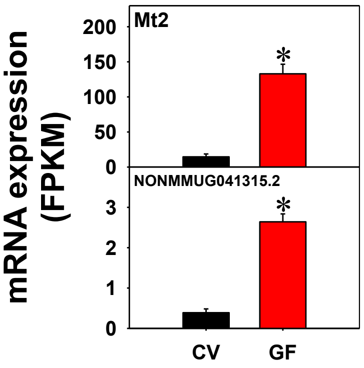

Supplementary Figure 4 Jejunum

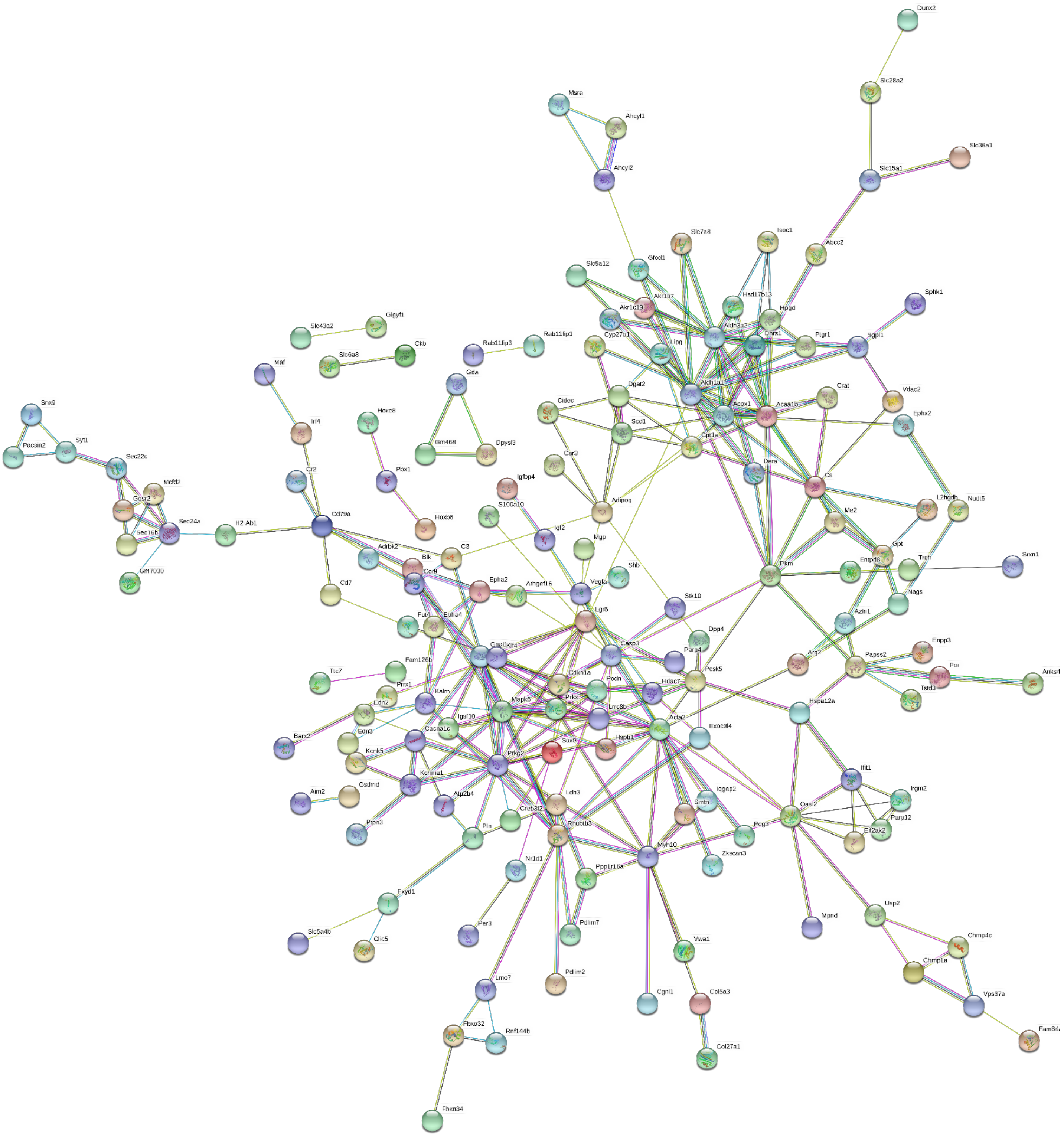

Supplementary Figure 5 Jejunum

A Drug metabolizing enzymes: Phase I

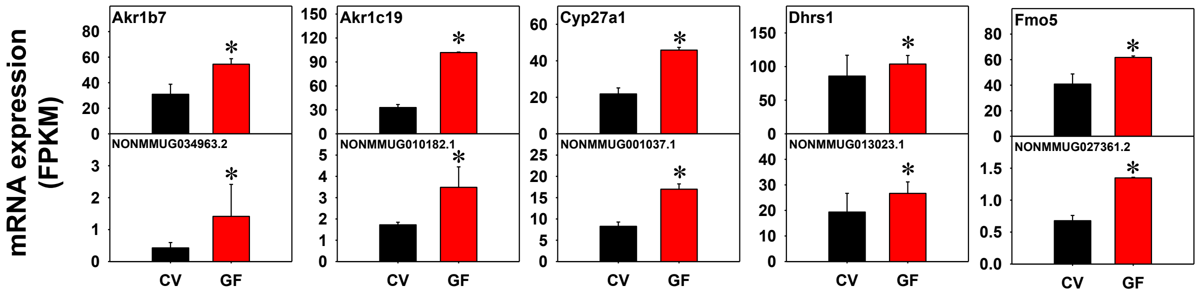

B Drug metabolizing enzymes: Phase II

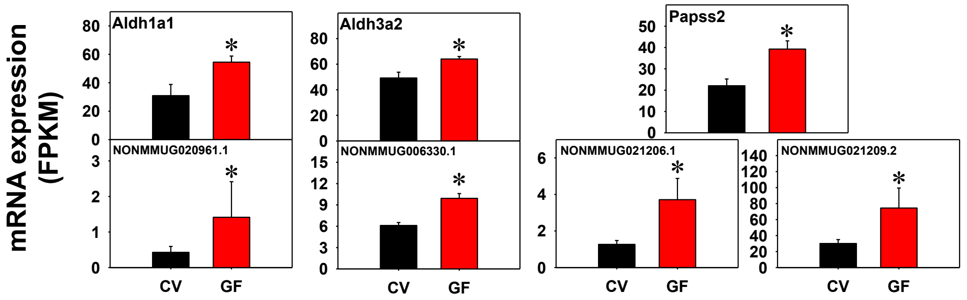

C Transporters

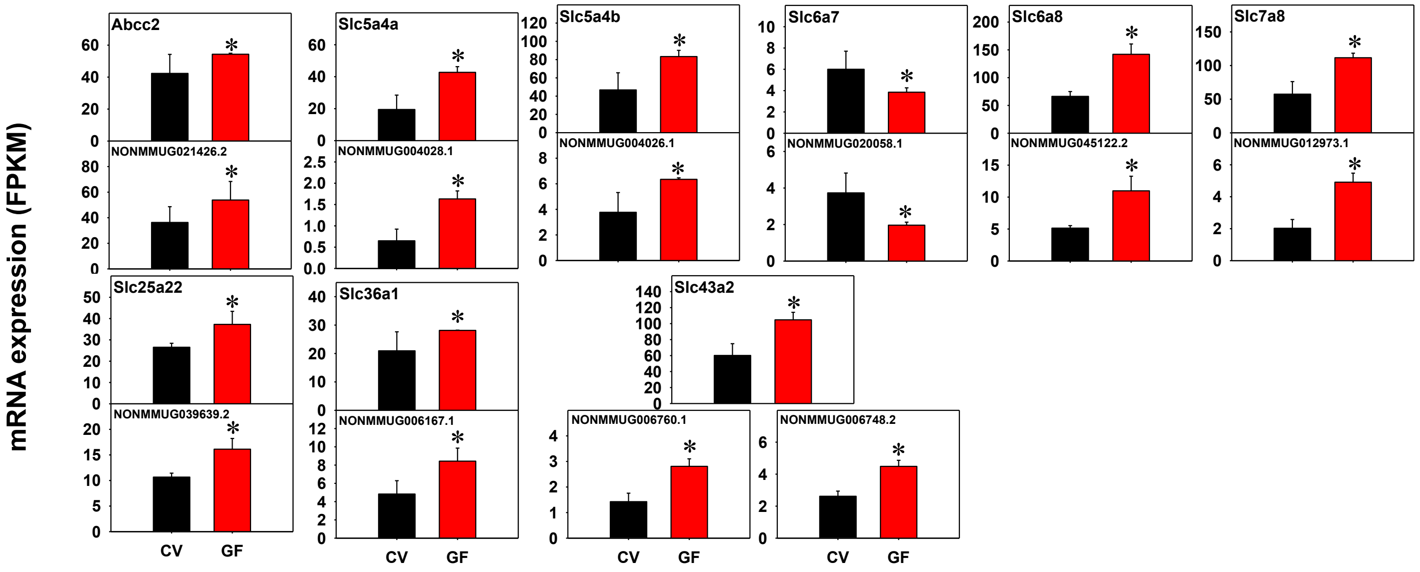

Supplementary Figure 6 Ileum

A Drug metabolizing enzymes: Phase I

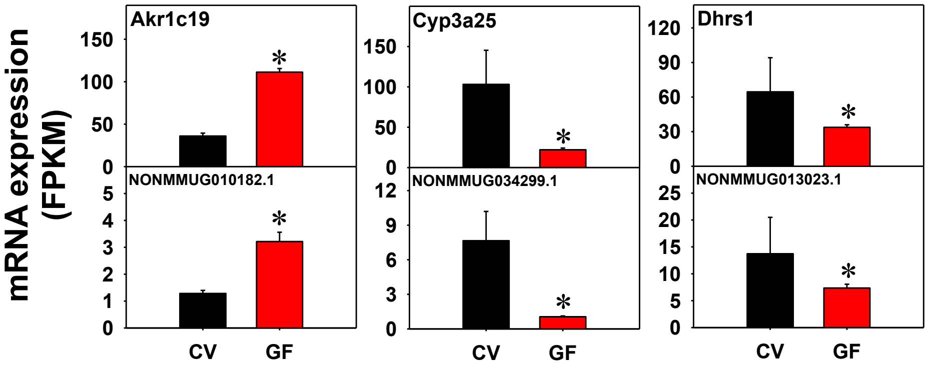

B Transporters

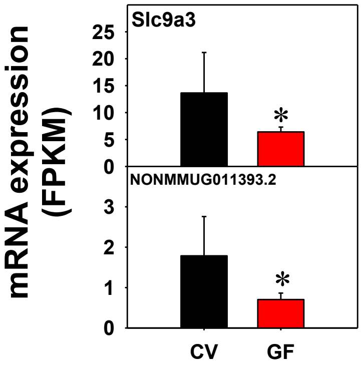

C Oxidative stress

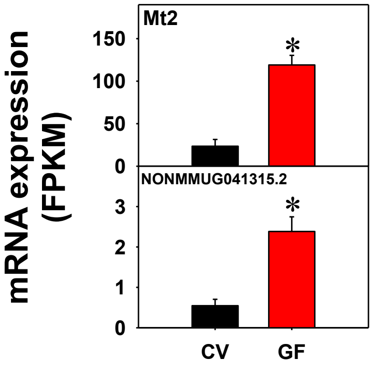

Supplementary Figure 7 Colon

A Drug metabolizing enzymes: Phase I

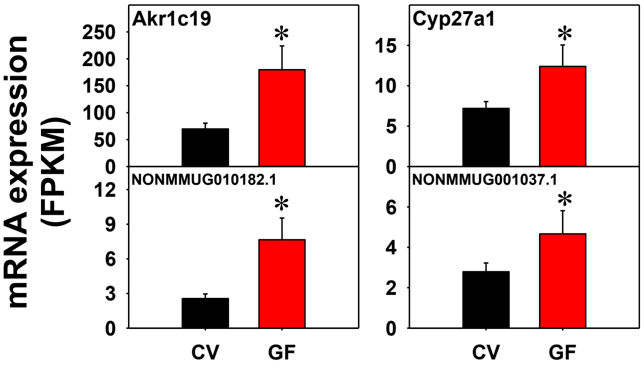

B Drug metabolizing enzymes: Phase II

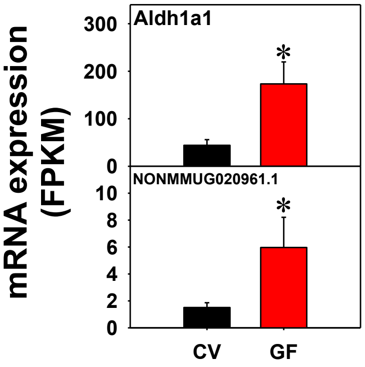

C Transporters

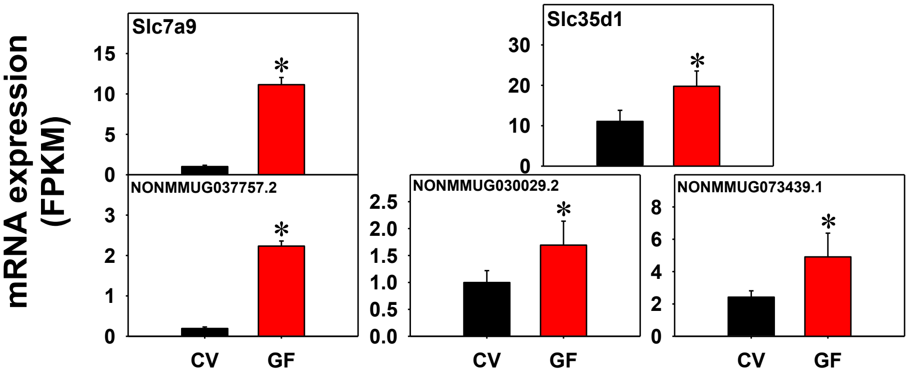

D Nuclear receptors

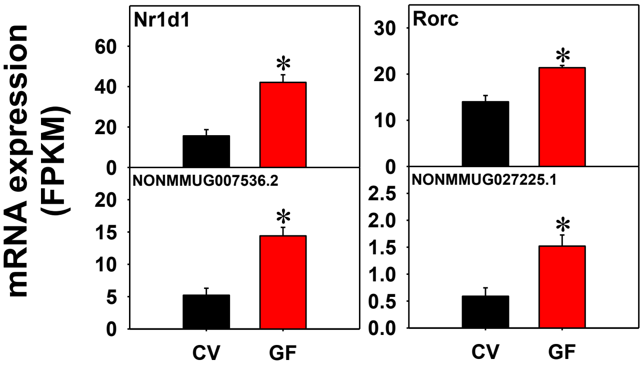

Supplementary Figure 8 BAT

lncRNA gene H19, imprinted maternally expressed transcript

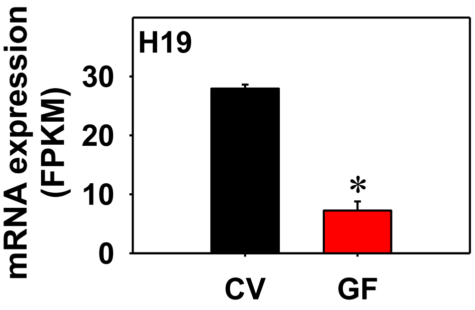

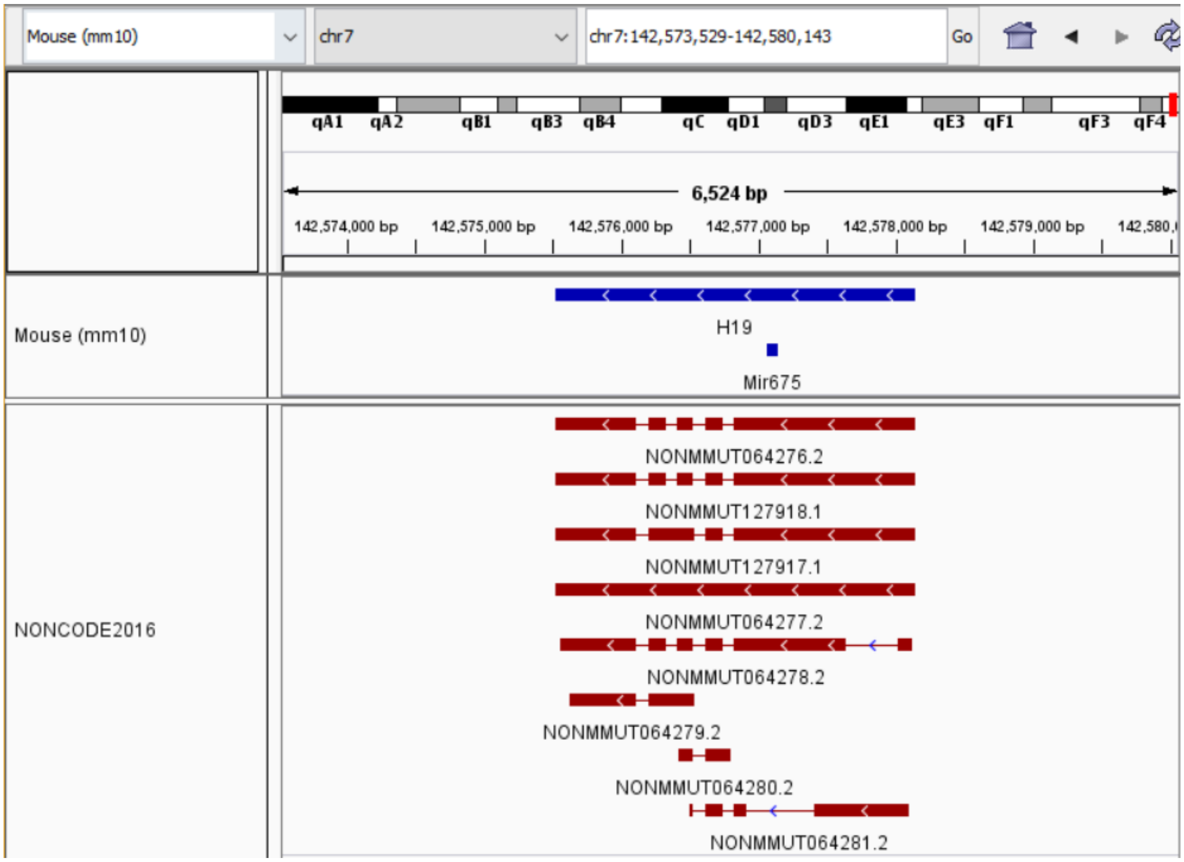

Supplementary Figure 9 Liver genomic annotations

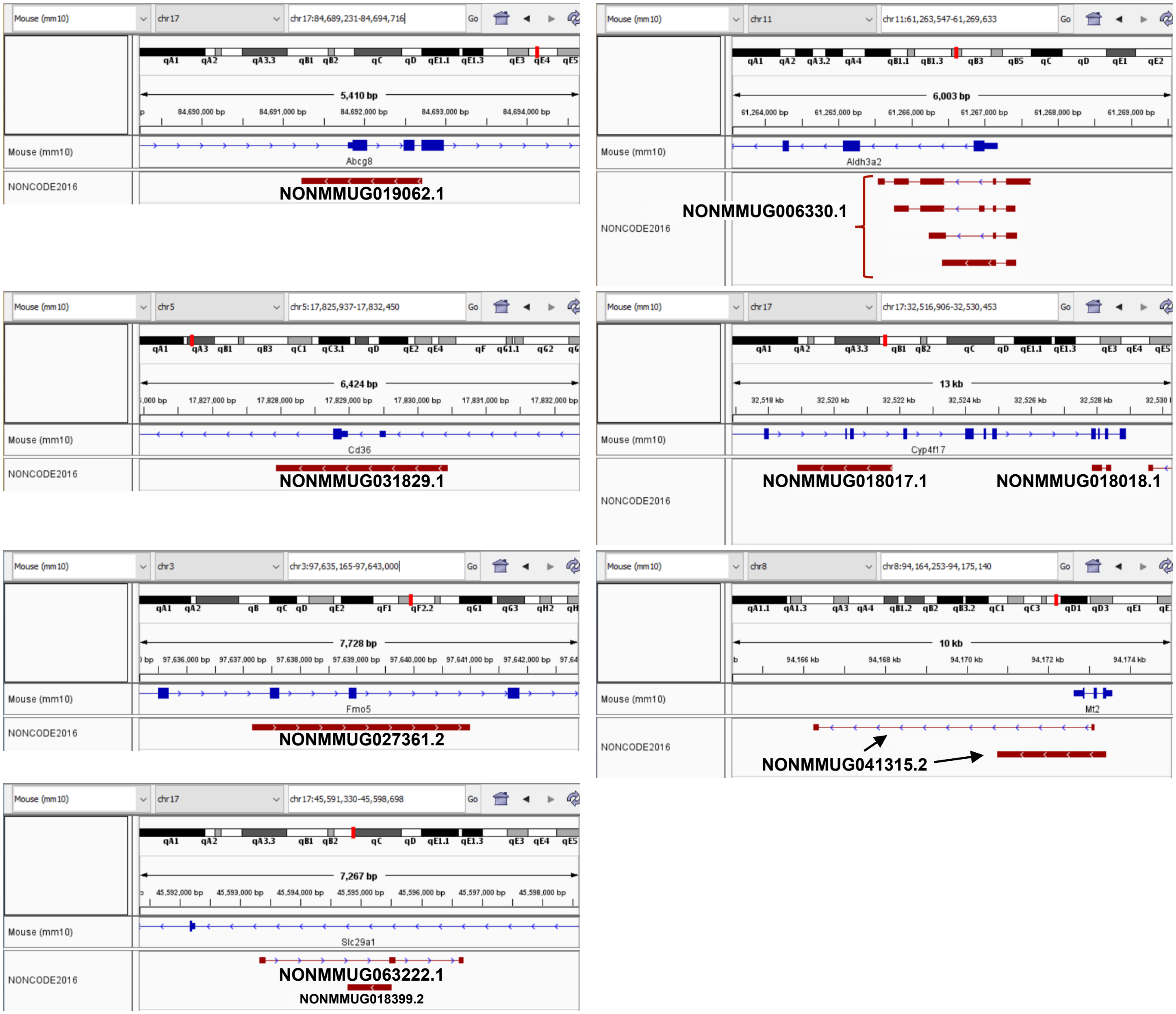

Supplementary Figure 9 Duodenum genomic annotations

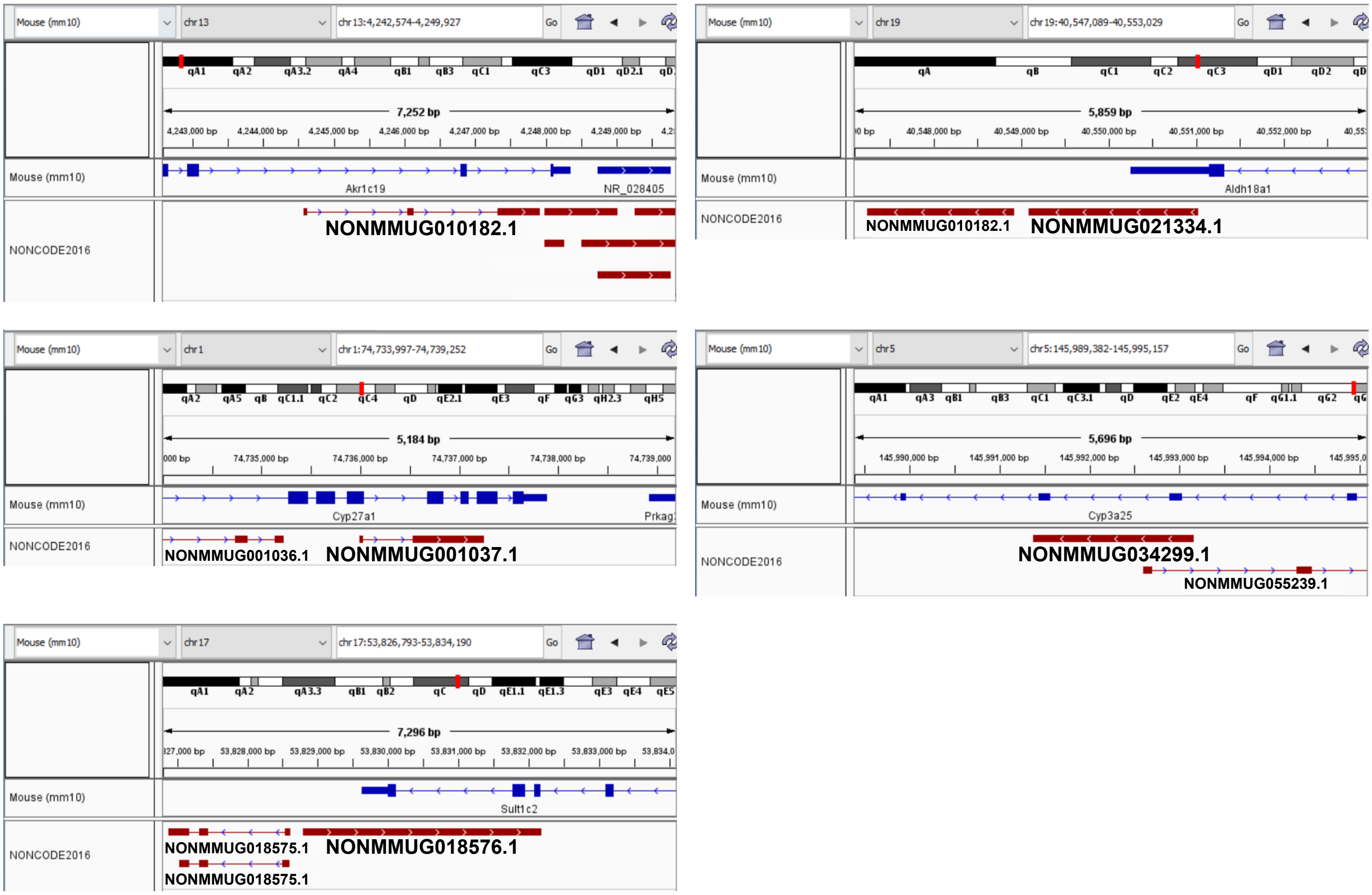

Supplementary Figure 9 Jejunum genomic annotations

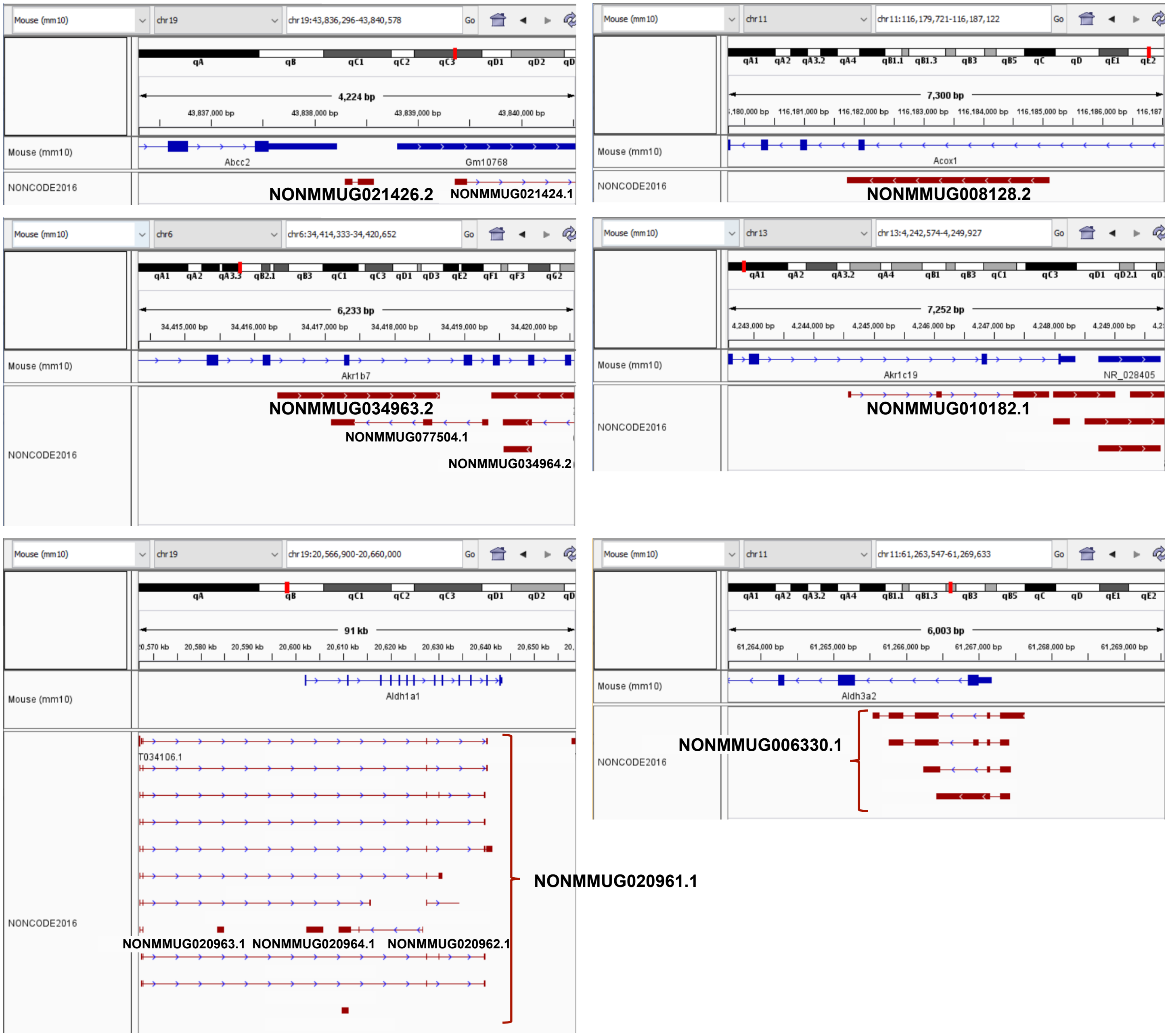

Supplementary Figure 9 Jejunum genomic annotations

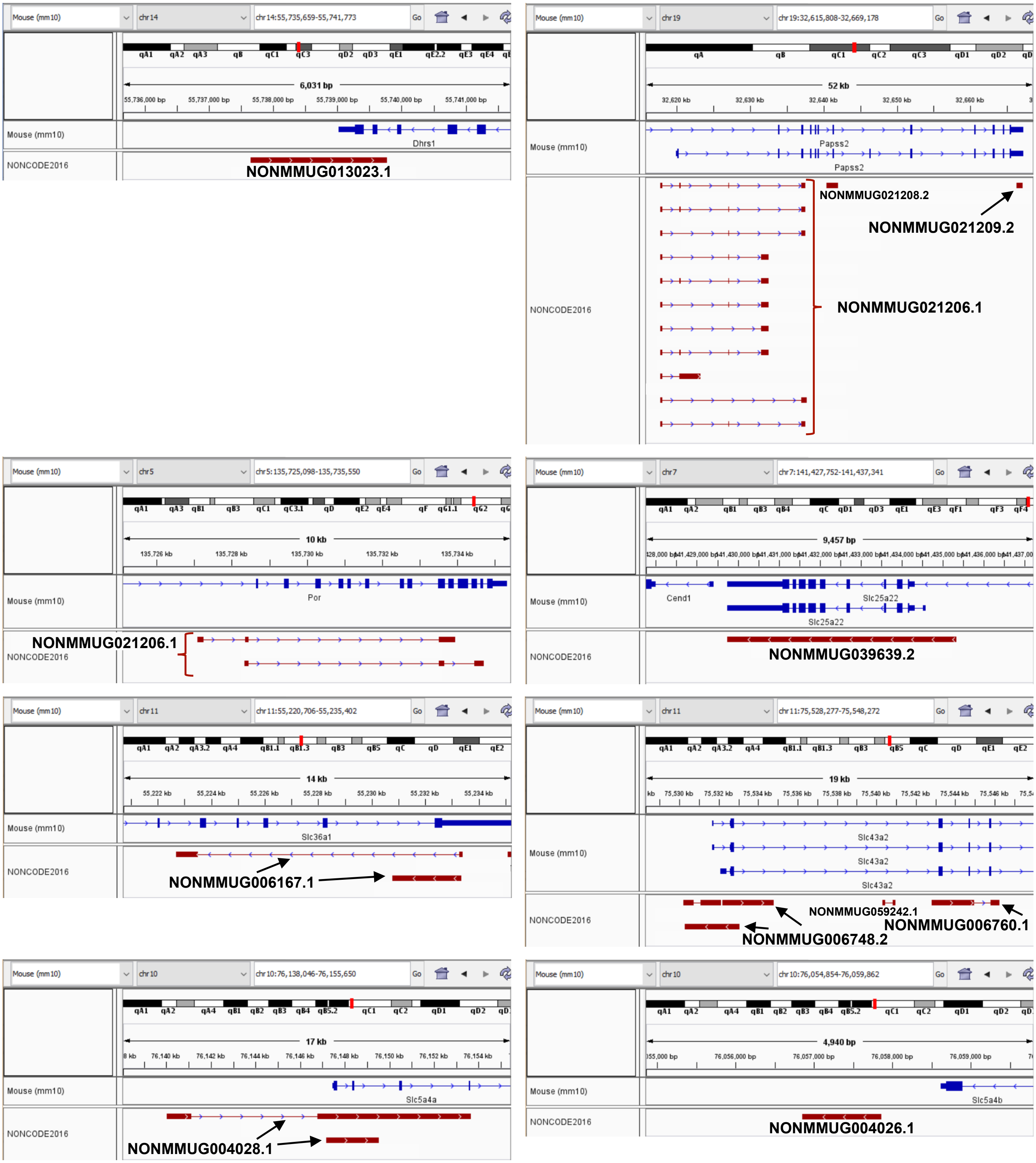

Supplementary Figure 9 Jejunum genomic annotations

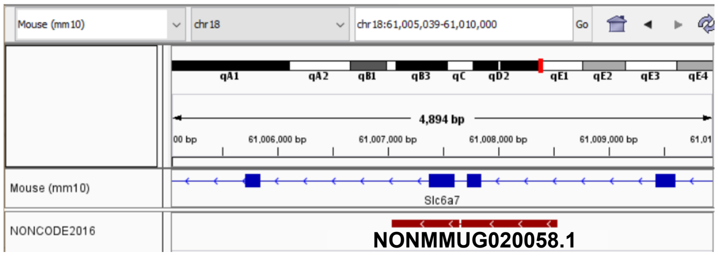

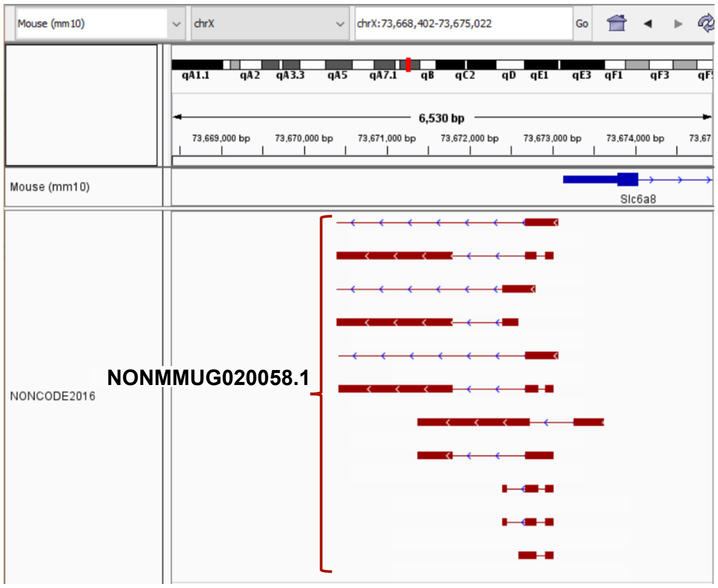

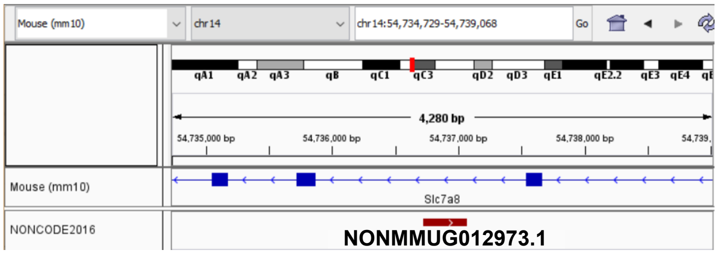

Supplementary Figure 9 Ileum and colon genomic annotations

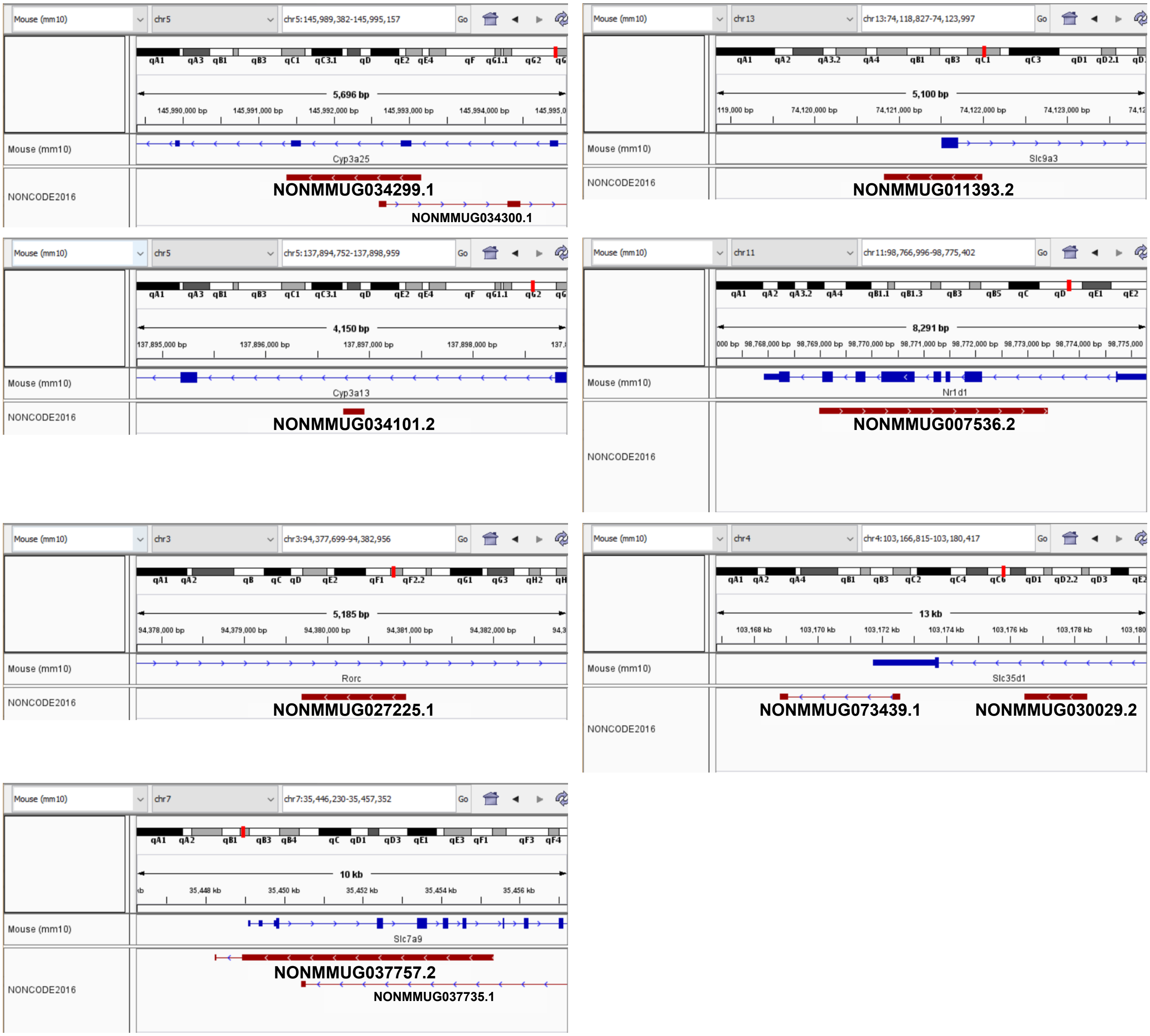

Supplemental Figure 10A Liver differentially regulated PCG network

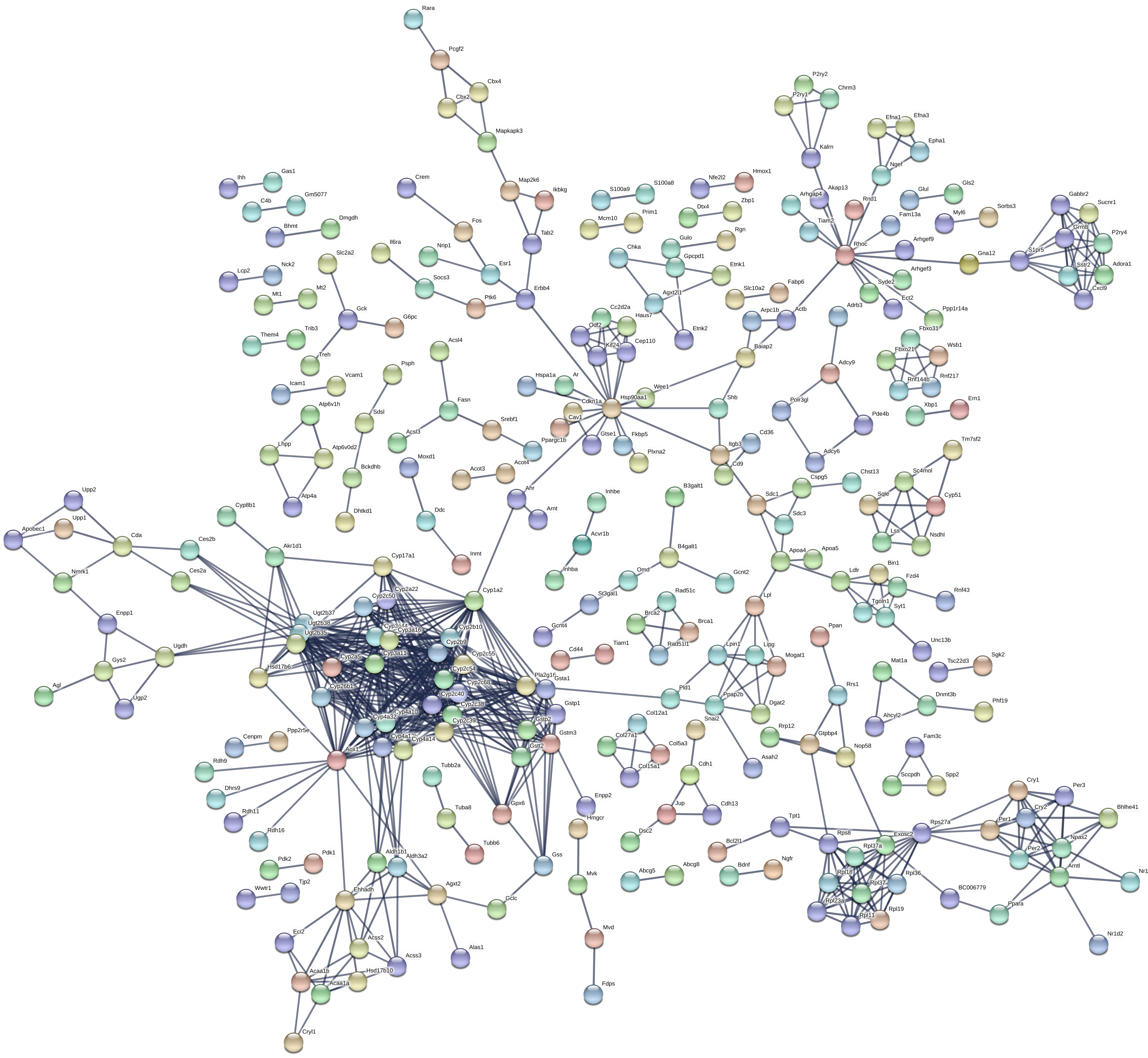

Supplemental Figure 10B Liver differentially regulated PCG network

Network Stats

number of nodes:	904	expected number of edges:	245
number of edges:	787	PPI enrichment p-value:	< 1.0e-16
average node degree:	1.74	*your network has significantly more interactions than expected (what does that mean?)*	
avg. local clustering coefficient:	0.251		

Functional enrichments in your network

	Biological Process (GO)		
pathway ID	*pathway description*	*count in gene set*	*false discovery rate*
GO:0006629	lipid metabolic process	100	1.13e-19
GO:0044699	single-organism process	507	1.13e-19
GO:0044281	small molecule metabolic process	126	1.78e-19
GO:0019752	carboxylic acid metabolic process	78	1.32e-15
GO:0044763	single-organism cellular process	458	1.32e-15

	Molecular Function (GO)		
pathway ID	*pathway description*	*count in gene set*	*false discovery rate*
GO:0005488	binding	474	2.02e-16
GO:0003674	molecular_function	535	3.37e-15
GO:0043167	ion binding	306	1.42e-14
GO:0003824	catalytic activity	291	6.32e-14
GO:0004497	monooxygenase activity	25	8.6e-11

	Cellular Component (GO)		
pathway ID	*pathway description*	*count in gene set*	*false discovery rate*
GO:0005575	cellular_component	690	1.02e-26
GO:0005623	cell	623	6.46e-22
GO:0044464	cell part	618	6.05e-21
GO:0005737	cytoplasm	483	2.01e-19
GO:0005622	intracellular	565	3.48e-18

	KEGG Pathways		
pathway ID	*pathway description*	*count in gene set*	*false discovery rate*
01100	Metabolic pathways	120	4.95e-18
00830	Retinol metabolism	26	8.22e-15
05204	Chemical carcinogenesis	22	3.86e-10
03320	PPAR signaling pathway	20	1.32e-09
00140	Steroid hormone biosynthesis	18	1.65e-07

	PFAM Protein Domains		
pathway ID	*pathway description*	*count in gene set*	*false discovery rate*
PF00067	Cytochrome P450	18	2.65e-09
PF00104	Ligand-binding domain of nuclear hormone receptor	7	0.049
PF00105	Zinc finger, C4 type (two domains)	7	0.049

	INTERPRO Protein Domains and Features		
pathway ID	*pathway description*	*count in gene set*	*false discovery rate*
IPR001128	Cytochrome P450	18	5.82e-09
IPR017972	Cytochrome P450, conserved site	17	1.77e-08
IPR002401	Cytochrome P450, E-class, group I	13	1.98e-05
IPR001628	Zinc finger, nuclear hormone receptor-type	7	0.0408
IPR001723	Nuclear hormone receptor	7	0.0408

Supplemental Figure 11A Duodenum differentially regulated PCG network

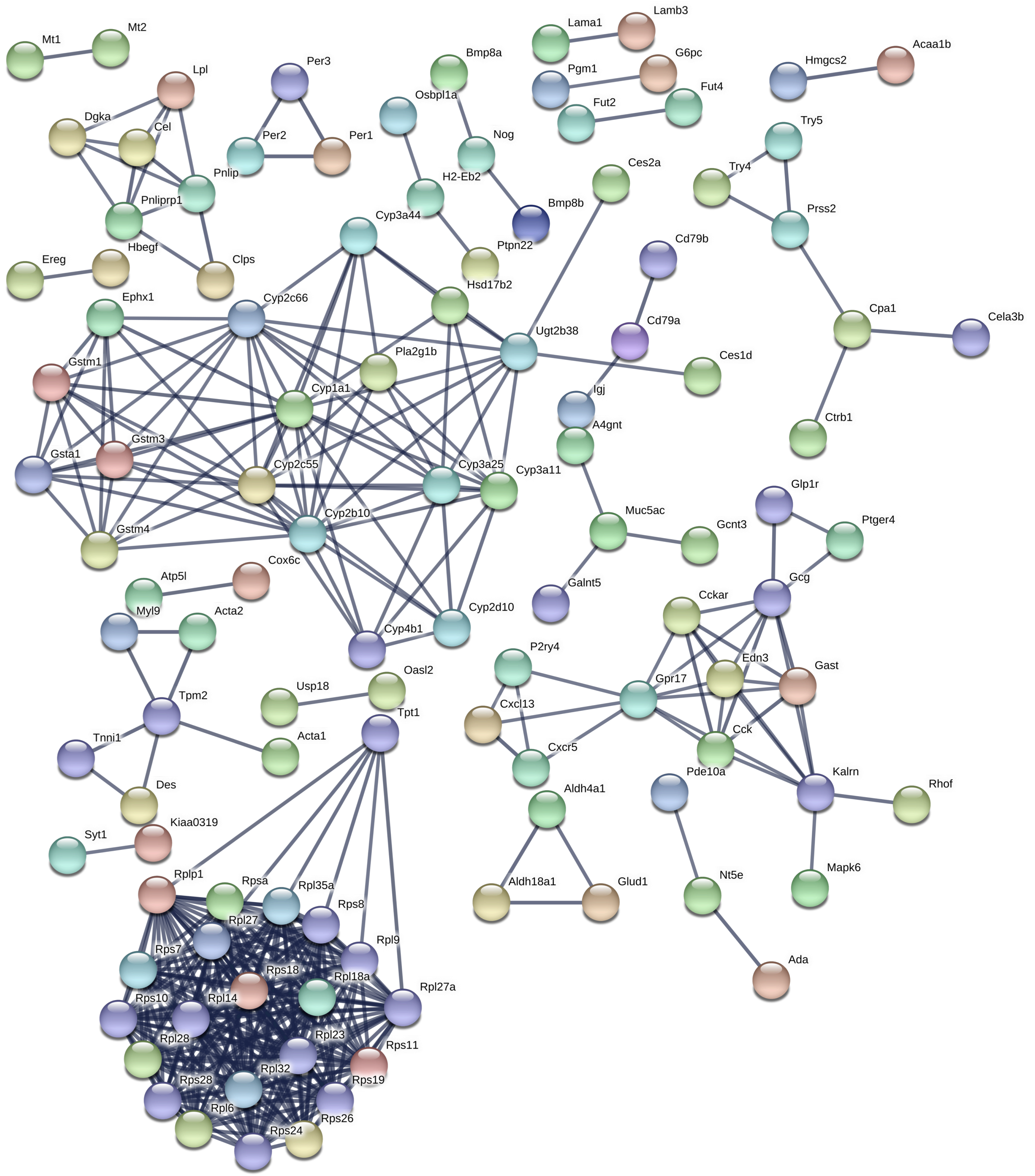

Supplemental Figure 11B Duodenum differentially regulated PCG network

Network Stats

number of nodes:	428	expected number of edges:	109
number of edges:	377	PPI enrichment p-value:	< 1.0e-16
average node degree:	1.76		
avg. local clustering coefficient:	0.232		

your network has significantly more interactions than expected (what does that mean?)

Functional enrichments in your network

Biological Process (GO)

pathway ID	*pathway description*	*count in gene set*	*false discovery rate*
GO:0006811	ion transport	39	0.00692
GO:0006814	sodium ion transport	13	0.00692
GO:0006820	anion transport	21	0.00692
GO:0042221	response to chemical	77	0.00692
GO:0042493	response to drug	22	0.00692

Molecular Function (GO)

pathway ID	*pathway description*	*count in gene set*	*false discovery rate*
GO:0003735	structural constituent of ribosome	17	7.55e-06
GO:0015293	symporter activity	15	3.73e-05
GO:0005198	structural molecule activity	27	6.99e-05
GO:0015291	secondary active transmembrane transporter activity	16	0.000624
GO:0003674	molecular_function	242	0.000681

Cellular Component (GO)

pathway ID	*pathway description*	*count in gene set*	*false discovery rate*
GO:0022626	cytosolic ribosome	18	3.13e-11
GO:0044391	ribosomal subunit	18	6.17e-08
GO:0044421	extracellular region part	111	6.17e-08
GO:0005576	extracellular region	120	3.59e-07
GO:0044444	cytoplasmic part	166	4.35e-07

KEGG Pathways

pathway ID	*pathway description*	*count in gene set*	*false discovery rate*
04972	Pancreatic secretion	18	1.64e-10
05204	Chemical carcinogenesis	13	1.76e-06
04978	Mineral absorption	9	1.58e-05
04974	Protein digestion and absorption	11	6.61e-05
00140	Steroid hormone biosynthesis	10	0.000188

Supplemental Figure 12A Jejunum differentially regulated PCG network

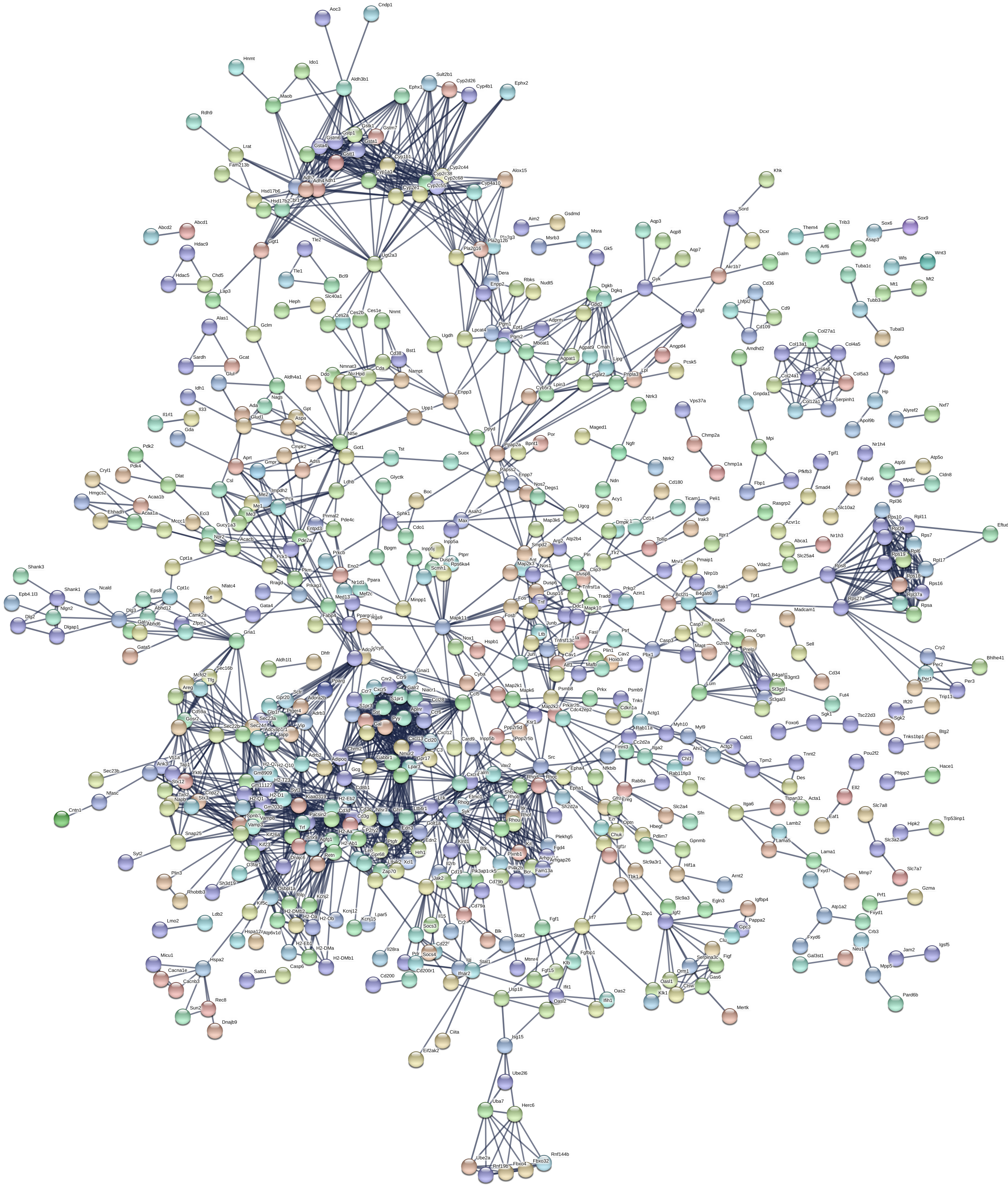

Supplemental Figure 12B Jejunum differentially regulated PCG network

Network Stats

number of nodes:	1790	expected number of edges:	909
number of edges:	2185	PPI enrichment p-value:	< 1.0e-16
average node degree:	2.44		
avg. local clustering coefficient:	0.27		

your network has significantly more interactions than expected (what does that mean?)

Functional enrichments in your network

Biological Process (GO)

pathway ID	*pathway description*	*count in gene set*	*false discovery rate*
GO:0044763	single-organism cellular process	899	3.71e-31
GO:0044699	single-organism process	962	8.83e-31
GO:0042221	response to chemical	343	6.45e-25
GO:0009987	cellular process	1021	1.13e-24
GO:0050896	response to stimulus	591	1.58e-24

Molecular Function (GO)

pathway ID	*pathway description*	*count in gene set*	*false discovery rate*
GO:0003674	molecular_function	1045	6.66e-29
GO:0005488	binding	873	7.68e-20
GO:0005515	protein binding	513	8.37e-20
GO:0043167	ion binding	520	3.42e-11
GO:0005102	receptor binding	138	3.38e-08

Cellular Component (GO)

pathway ID	*pathway description*	*count in gene set*	*false discovery rate*
GO:0005575	cellular_component	1307	3.65e-36
GO:0005886	plasma membrane	481	8.19e-35
GO:0016020	membrane	804	1.28e-34
GO:0071944	cell periphery	487	1.49e-34
GO:0044425	membrane part	644	4.2e-34

KEGG Pathways

pathway ID	*pathway description*	*count in gene set*	*false discovery rate*
05168	Herpes simplex infection	52	9.33e-13
05164	Influenza A	43	4.24e-10
05145	Toxoplasmosis	31	3.65e-08
04640	Hematopoietic cell lineage	25	1.5e-07
04974	Protein digestion and absorption	26	1.59e-07

PFAM Protein Domains

pathway ID	*pathway description*	*count in gene set*	*false discovery rate*
PF00969	Class II histocompatibility antigen, beta domain	6	0.000668
PF07654	Immunoglobulin C1-set domain	15	0.00134
PF00879	Defensin propeptide	8	0.0471

INTERPRO Protein Domains and Features

pathway ID	*pathway description*	*count in gene set*	*false discovery rate*
IPR014745	MHC class II, alpha/beta chain, N-terminal	8	9.09e-06
IPR003006	Immunoglobulin/major histocompatibility complex, conserved site	15	0.000147
IPR000353	MHC class II, beta chain, N-terminal	6	0.000367
IPR011162	MHC classes I/II-like antigen recognition protein	15	0.000367
IPR003597	Immunoglobulin C1-set	15	0.00118

Supplemental Figure 13A Ileum differentially regulated PCG network

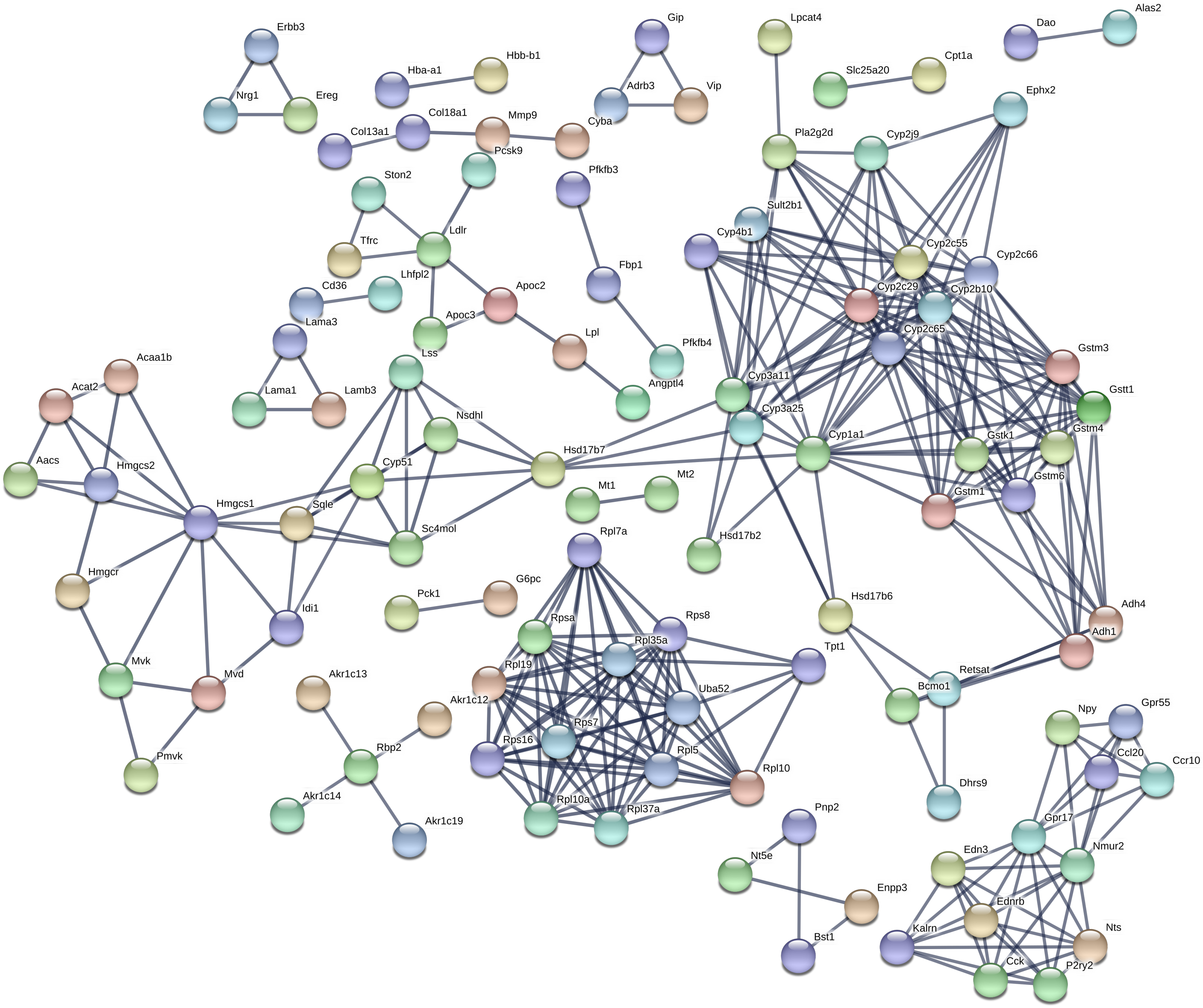

Supplemental Figure 13B Ileum differentially regulated PCG network

Network Stats

number of nodes:	359	expected number of edges:	75
number of edges:	333	PPI enrichment p-value:	< 1.0e-16
average node degree:	1.86		
avg. local clustering coefficient:	0.243		

your network has significantly more interactions than expected (what does that mean?)

Functional enrichments in your network

Biological Process (GO)

pathway ID	*pathway description*	*count in gene set*	*false discovery rate*
GO:0006629	lipid metabolic process	55	1.55e-15
GO:0008202	steroid metabolic process	26	1.32e-13
GO:0044255	cellular lipid metabolic process	40	1.67e-10
GO:1901615	organic hydroxy compound metabolic process	30	1.67e-10
GO:0006066	alcohol metabolic process	25	2.83e-10

Molecular Function (GO)

pathway ID	*pathway description*	*count in gene set*	*false discovery rate*
GO:0003824	catalytic activity	117	8.43e-05
GO:0004497	monooxygenase activity	12	8.43e-05
GO:0016491	oxidoreductase activity	31	8.43e-05
GO:0015293	symporter activity	12	0.000871
GO:0016705	oxidoreductase activity, acting on paired donors, with incorporation or reduction of molecular oxygen	13	0.000871

Cellular Component (GO)

pathway ID	*pathway description*	*count in gene set*	*false discovery rate*
GO:0005576	extracellular region	114	7.31e-10
GO:0044421	extracellular region part	102	1.22e-09
GO:0005615	extracellular space	45	1.54e-06
GO:0031224	intrinsic component of membrane	118	1.54e-06
GO:0065010	extracellular membrane-bounded organelle	74	5e-06

KEGG Pathways

pathway ID	*pathway description*	*count in gene set*	*false discovery rate*
05204	Chemical carcinogenesis	16	1.97e-10
00830	Retinol metabolism	14	5.73e-09
03320	PPAR signaling pathway	13	2.62e-08
00900	Terpenoid backbone biosynthesis	8	4.42e-08
01100	Metabolic pathways	49	1.02e-07

PFAM Protein Domains

pathway ID	*pathway description*	*count in gene set*	*false discovery rate*
PF00067	Cytochrome P450	8	0.00484
PF00879	Defensin propeptide	5	0.013

INTERPRO Protein Domains and Features

pathway ID	*pathway description*	*count in gene set*	*false discovery rate*
IPR001128	Cytochrome P450	8	0.00531
IPR017972	Cytochrome P450, conserved site	8	0.00531
IPR002366	Defensin propeptide	5	0.0143
IPR016327	Alpha-defensin	5	0.0143

Supplemental Figure 14A Colon differentially regulated PCG network

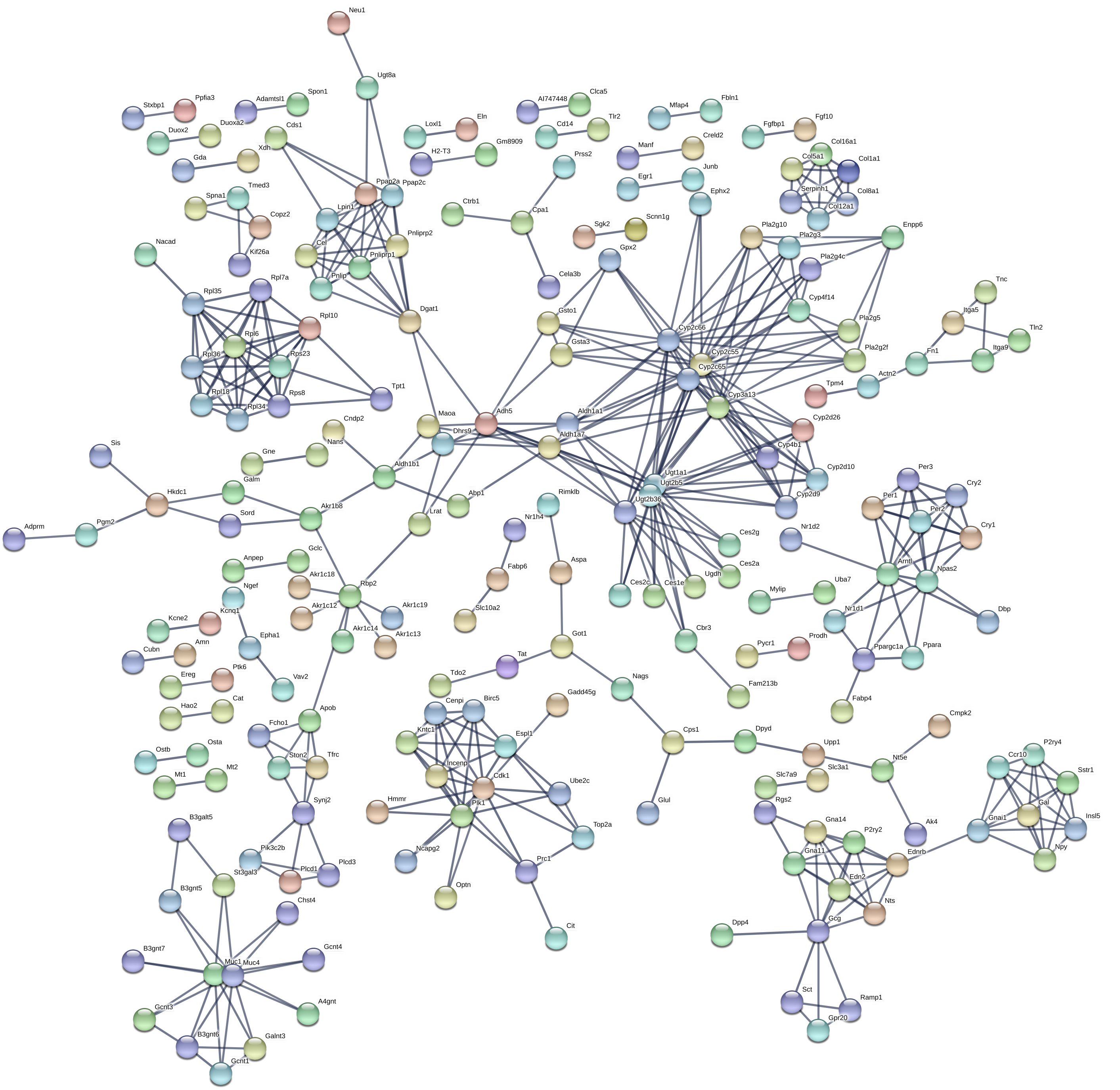

Supplemental Figure 14B Colon differentially regulated PCG network

Network Stats

number of nodes:	811	expected number of edges:	157
number of edges:	471	PPI enrichment p-value:	< 1.0e-16
average node degree:	1.16		
avg. local clustering coefficient:	0.208		

your network has significantly more interactions than expected (what does that mean?)

Functional enrichments in your network

Biological Process (GO)

pathway ID	*pathway description*	*count in gene set*	*false discovery rate*
GO:0044763	single-organism cellular process	409	1.46e-12
GO:0044699	single-organism process	437	1.97e-12
GO:0009987	cellular process	462	2.11e-09
GO:0006820	anion transport	43	7.54e-09
GO:0050896	response to stimulus	263	1.92e-08

Molecular Function (GO)

pathway ID	*pathway description*	*count in gene set*	*false discovery rate*
GO:0003674	molecular_function	461	1.43e-08
GO:0005488	binding	394	2.33e-07
GO:0005515	protein binding	227	1.74e-06
GO:0005215	transporter activity	68	3.01e-05
GO:0008509	anion transmembrane transporter activity	25	3.14e-05

Cellular Component (GO)

pathway ID	*pathway description*	*count in gene set*	*false discovery rate*
GO:0005576	extracellular region	251	2.72e-22
GO:0005575	cellular_component	608	3.83e-20
GO:0044421	extracellular region part	215	3.03e-18
GO:0016020	membrane	363	3.39e-14
GO:0070062	extracellular exosome	163	1.03e-13

KEGG Pathways

pathway ID	*pathway description*	*count in gene set*	*false discovery rate*
04974	Protein digestion and absorption	26	1.16e-14
01100	Metabolic pathways	93	4.34e-10
04975	Fat digestion and absorption	14	3.13e-09
04972	Pancreatic secretion	20	2.89e-08
04710	Circadian rhythm	9	2.74e-05

PFAM Protein Domains

pathway ID	*pathway description*	*count in gene set*	*false discovery rate*
PF00104	Ligand-binding domain of nuclear hormone receptor	7	0.0368
PF00105	Zinc finger, C4 type (two domains)	7	0.0368

Supplemental Figure 15A BAT differentially regulated PCG network

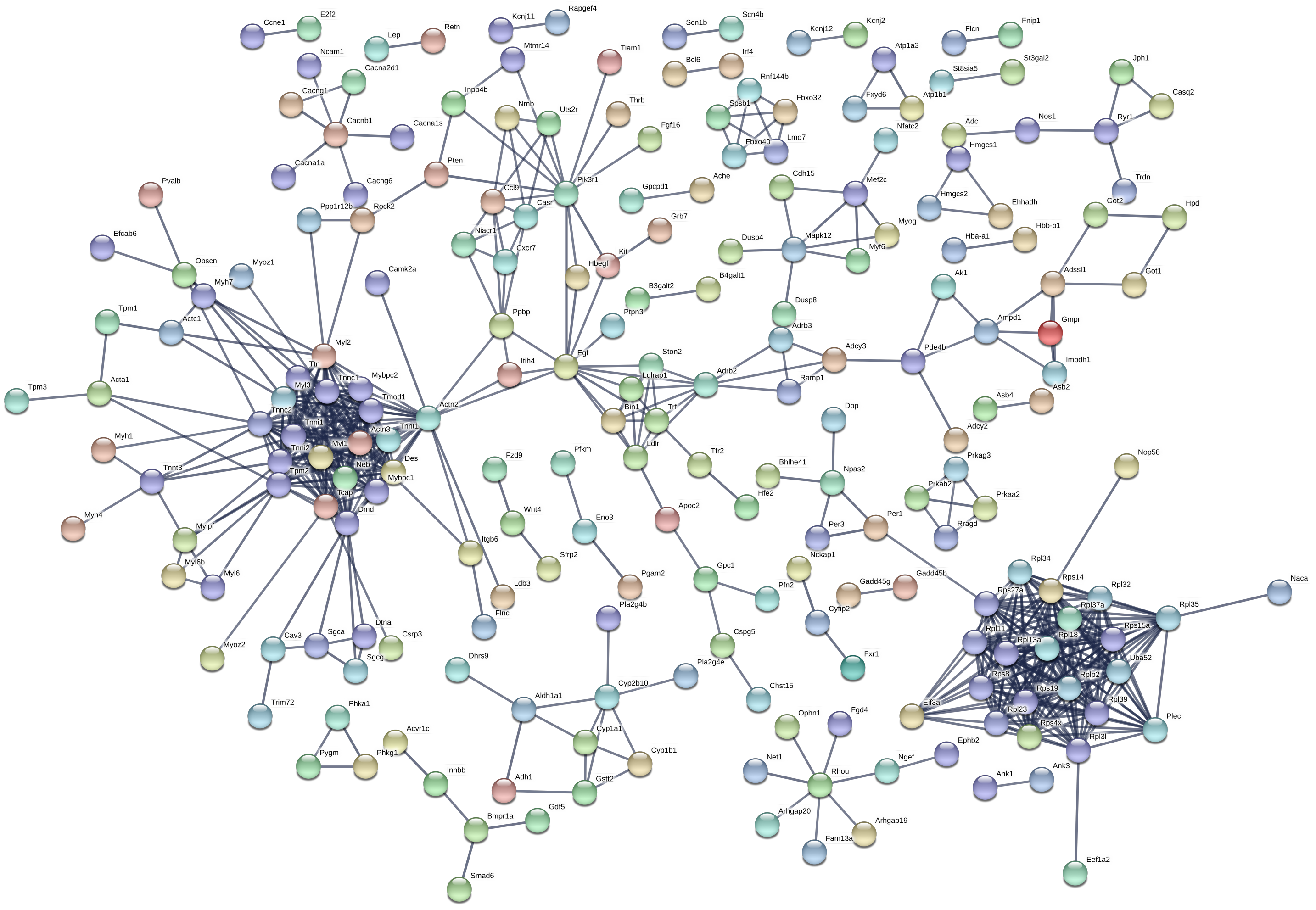

Supplemental Figure 15B BAT differentially regulated PCG network

Network Stats

number of nodes: 756	expected number of edges: 163
number of edges: 590	PPI enrichment p-value: < 1.0e-16
average node degree: 1.56	*your network has significantly more interactions than expected (what does that mean?)*
avg. local clustering coefficient: 0.235	

Functional enrichments in your network

Biological Process (GO)

pathway ID	*pathway description*	*count in gene set*	*false discovery rate*
GO:0061061	muscle structure development	76	2.89e-31
GO:0003012	muscle system process	50	8.75e-31
GO:0006936	muscle contraction	41	5.32e-26
GO:0044707	single-multicellular organism process	280	6.6e-24
GO:0065007	biological regulation	404	1.22e-22

Molecular Function (GO)

pathway ID	*pathway description*	*count in gene set*	*false discovery rate*
GO:0008092	cytoskeletal protein binding	74	1.21e-23
GO:0005515	protein binding	258	5.52e-19
GO:0005488	binding	403	1.23e-15
GO:0003674	molecular_function	455	1.06e-14
GO:0008307	structural constituent of muscle	11	1.61e-10

Cellular Component (GO)

pathway ID	*pathway description*	*count in gene set*	*false discovery rate*
GO:0030016	myofibril	75	1.62e-60
GO:0043292	contractile fiber	76	2.13e-60
GO:0044449	contractile fiber part	70	4.51e-57
GO:0030017	sarcomere	68	2.72e-56
GO:0031674	I band	46	7.94e-36

KEGG Pathways

pathway ID	*pathway description*	*count in gene set*	*false discovery rate*
05410	Hypertrophic cardiomyopathy (HCM)	22	6.89e-12
05414	Dilated cardiomyopathy	21	1.27e-10
04921	Oxytocin signaling pathway	25	5.28e-09
04261	Adrenergic signaling in cardiomyocytes	23	2.1e-08
04260	Cardiac muscle contraction	16	1.55e-07

PFAM Protein Domains

pathway ID	*pathway description*	*count in gene set*	*false discovery rate*
PF00307	Calponin homology (CH) domain	7	0.0136
PF00041	Fibronectin type III domain	8	0.0305
PF03250	Tropomodulin	3	0.0305
PF07679	Immunoglobulin I-set domain	10	0.0305

INTERPRO Protein Domains and Features

pathway ID	*pathway description*	*count in gene set*	*false discovery rate*
IPR001715	Calponin homology domain	8	0.0114
IPR003961	Fibronectin type III	12	0.013

Supplemental Figure 16A WAT differentially regulated PCG network

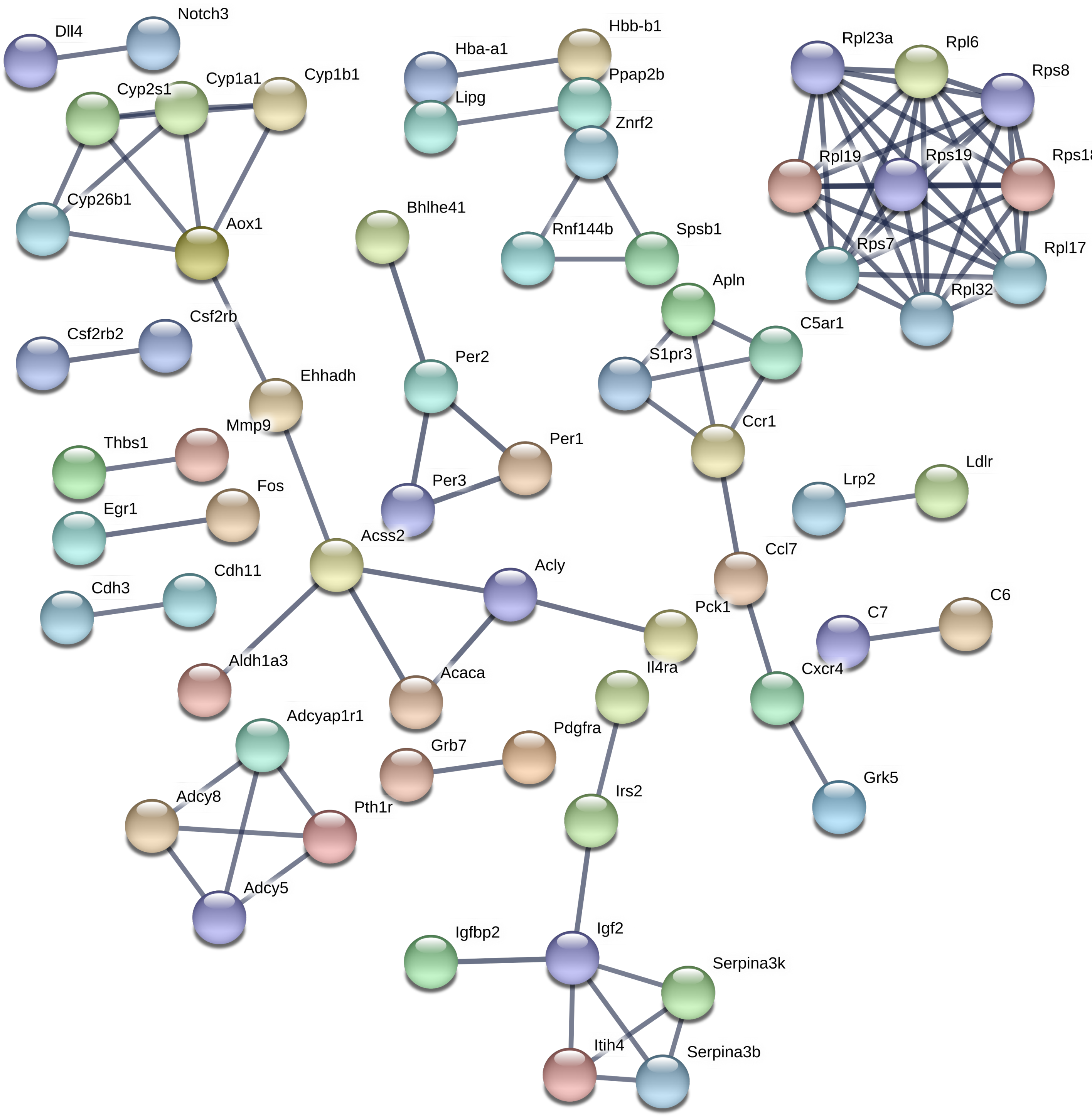

Supplemental Figure 16B WAT differentially regulated PCG network

Network Stats

number of nodes:	261	expected number of edges:	40
number of edges:	93	PPI enrichment p-value:	5.76e-13
average node degree:	0.713		
avg. local clustering coefficient:	0.218		

your network has significantly more interactions than expected (what does that mean?)

Functional enrichments in your network

Biological Process (GO)

pathway ID	*pathway description*	*count in gene set*	*false discovery rate*
GO:0007166	cell surface receptor signaling pathway	45	5.31e-07
GO:0010647	positive regulation of cell communication	44	5.31e-07
GO:0048522	positive regulation of cellular process	86	5.31e-07
GO:0007165	signal transduction	73	9.36e-07
GO:0023056	positive regulation of signaling	41	9.36e-07

Molecular Function (GO)

pathway ID	*pathway description*	*count in gene set*	*false discovery rate*
GO:0005198	structural molecule activity	24	7.66e-07
GO:0005515	protein binding	91	5.95e-06
GO:0005488	binding	140	0.000165
GO:0097110	scaffold protein binding	6	0.00325
GO:0019899	enzyme binding	34	0.00503

Cellular Component (GO)

pathway ID	*pathway description*	*count in gene set*	*false discovery rate*
GO:0044421	extracellular region part	80	9.72e-09
GO:0005615	extracellular space	38	7.8e-07
GO:0005575	cellular_component	198	1.27e-06
GO:0005576	extracellular region	81	1.27e-06
GO:0071944	cell periphery	79	1.27e-06

KEGG Pathways

pathway ID	*pathway description*	*count in gene set*	*false discovery rate*
04713	Circadian entrainment	8	0.00345
04670	Leukocyte transendothelial migration	8	0.0116
00980	Metabolism of xenobiotics by cytochrome P450	5	0.0282
01120	Microbial metabolism in diverse environments	8	0.0282
04710	Circadian rhythm	4	0.0282

PFAM Protein Domains

pathway ID	*pathway description*	*count in gene set*	*false discovery rate*
PF00038	Intermediate filament protein	7	2.01e-05
PF00090	Thrombospondin type 1 domain	6	0.000345

INTERPRO Protein Domains and Features

pathway ID	*pathway description*	*count in gene set*	*false discovery rate*
IPR018039	Intermediate filament protein, conserved site	7	1.03e-05
IPR001664	Intermediate filament protein	7	2.21e-05
IPR000884	Thrombospondin type-1 (TSP1) repeat	6	0.00108
IPR002957	Keratin, type I	4	0.00177

Supplemental Figure 17A Muscle differentially regulated PCG network

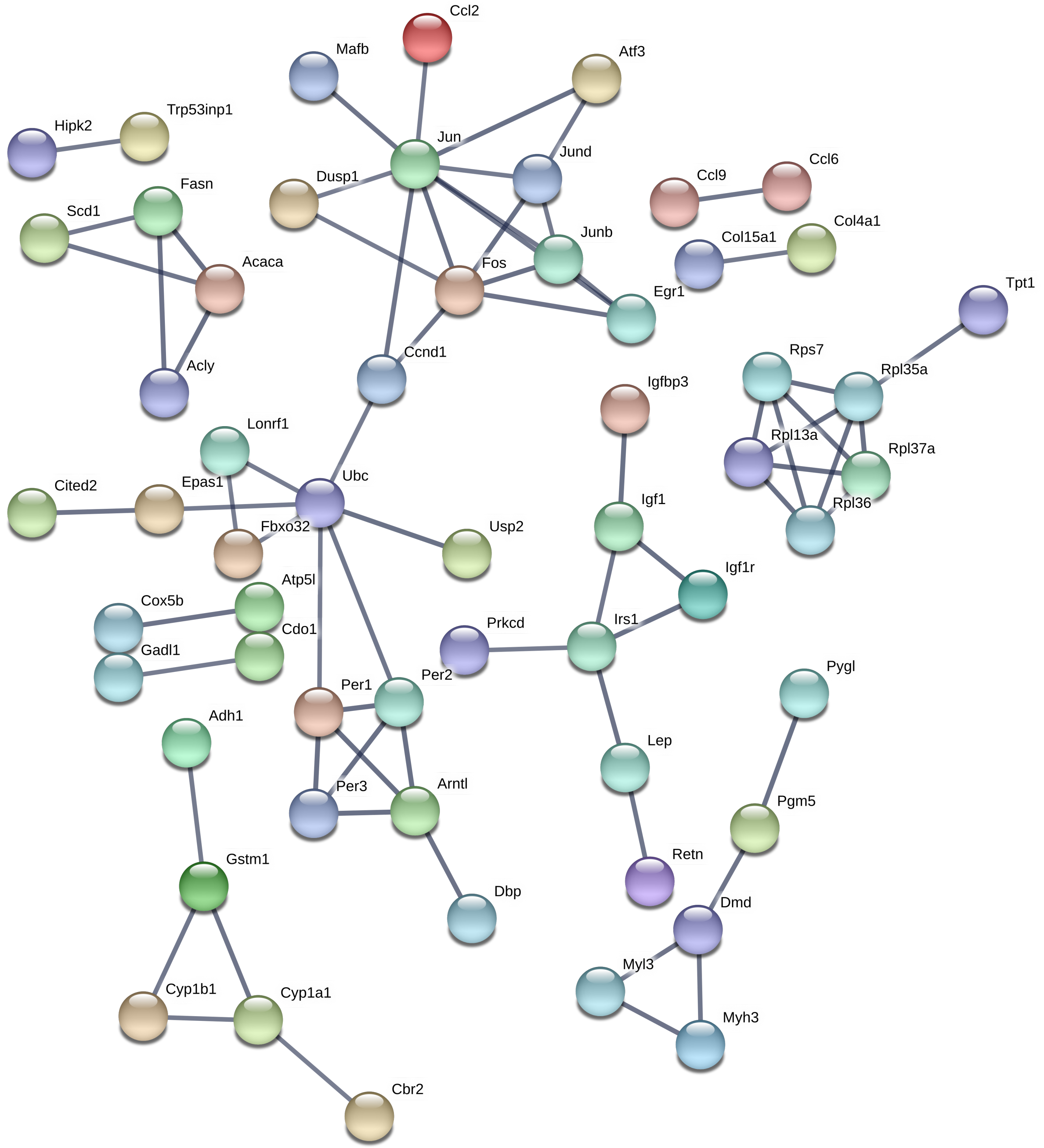

Supplemental Figure 17B Muscle differentially regulated PCG network

Network Stats

number of nodes:	196	expected number of edges:	22
number of edges:	71	PPI enrichment p-value:	1.11e-16
average node degree:	0.724	*your network has significantly more interactions than expected (what does that mean?)*	
avg. local clustering coefficient:	0.234		

Functional enrichments in your network

Biological Process (GO)

pathway ID	*pathway description*	*count in gene set*	*false discovery rate*
GO:0007623	circadian rhythm	17	2.82e-10
GO:0031324	negative regulation of cellular metabolic process	49	9.78e-09
GO:0048519	negative regulation of biological process	72	9.78e-09
GO:0070887	cellular response to chemical stimulus	43	1.07e-08
GO:0009892	negative regulation of metabolic process	51	1.69e-08

Molecular Function (GO)

pathway ID	*pathway description*	*count in gene set*	*false discovery rate*
GO:0005515	protein binding	80	1.6e-08
GO:0008134	transcription factor binding	17	0.000756
GO:0000982	transcription factor activity, RNA polymerase II core promoter proximal region sequence-specific binding	13	0.00605
GO:0019899	enzyme binding	28	0.00712
GO:0005488	binding	104	0.00735

Cellular Component (GO)

pathway ID	*pathway description*	*count in gene set*	*false discovery rate*
GO:0043292	contractile fiber	13	1.19e-05
GO:0044449	contractile fiber part	12	1.43e-05
GO:0030016	myofibril	12	2.25e-05
GO:0030017	sarcomere	11	4.13e-05
GO:0031012	extracellular matrix	15	0.000187

KEGG Pathways

pathway ID	*pathway description*	*count in gene set*	*false discovery rate*
04152	AMPK signaling pathway	10	4.26e-05
04068	FoxO signaling pathway	8	0.003
04710	Circadian rhythm	4	0.0103
00980	Metabolism of xenobiotics by cytochrome P450	5	0.015
04115	p53 signaling pathway	5	0.015

PFAM Protein Domains

pathway ID	*pathway description*	*count in gene set*	*false discovery rate*
PF05049	Interferon-inducible GTPase (IIGP)	4	0.0141

INTERPRO Protein Domains and Features

pathway ID	*pathway description*	*count in gene set*	*false discovery rate*
IPR007743	Immunity-related GTPases-like	4	0.0155
IPR030385	IRG-type guanine nucleotide-binding (G) domain	4	0.0155

Supplemental Figure 18. RT-qPCR validation of selected lncRNAs

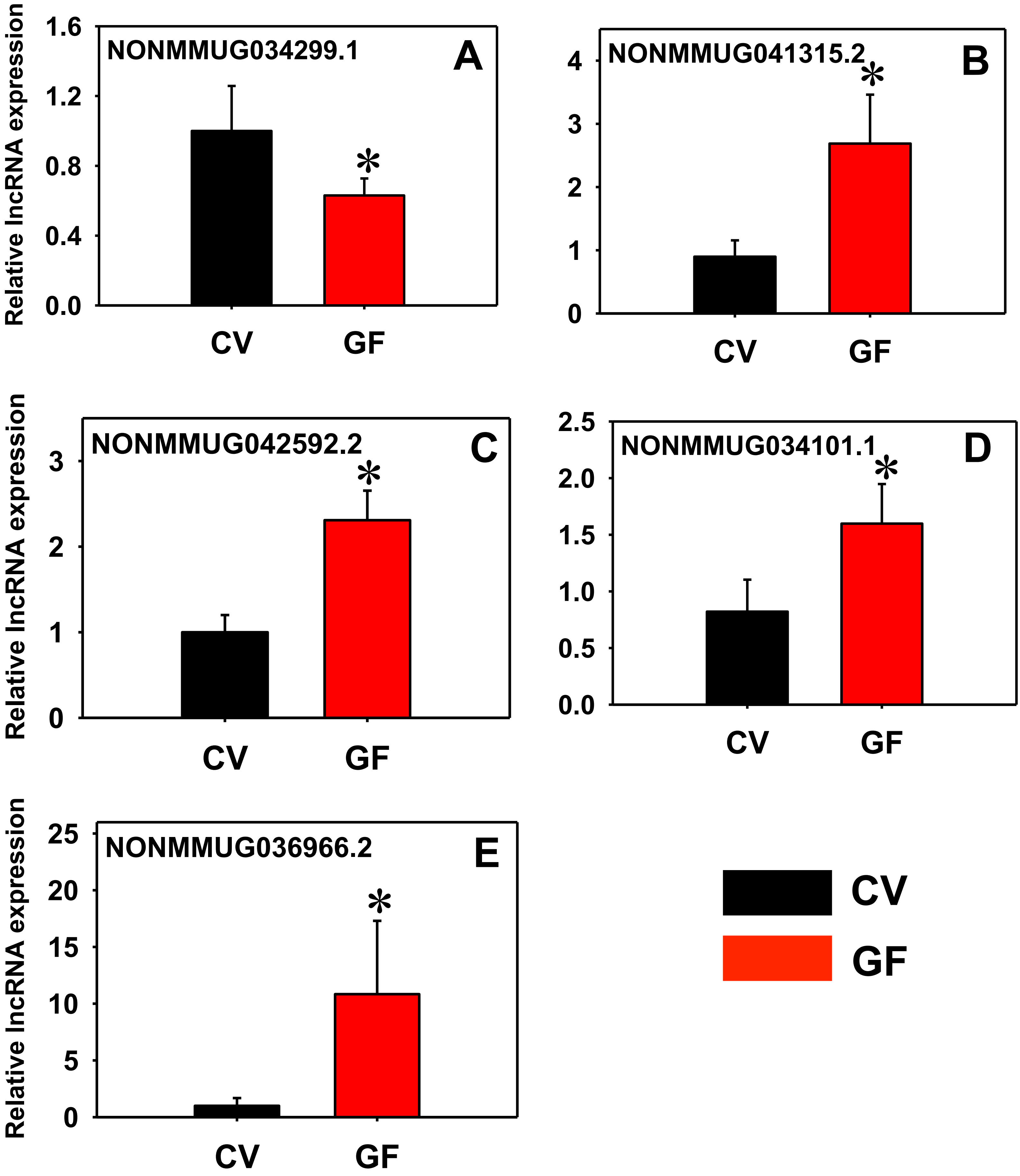

Supplement: Supplementary file 2 — Figure S1. Two-way hierarchical clustering dendrograms of differentially expressed lncRNAs by lack of gut microbiota. Figure S2-S8. Other examples of the expression of lncRNA-PCG pairs that were differentially regulated by lack of gut microbiota in liver (Figure S2), duodenum (Figure S3), jejunum (Figure S4-5), ileum (Figure S6), colon (Figure S7), and BAT (Figure S8). Figure S9. Genomic locations of lncRNA-PCG pairs described in Additional file 2: Figures S2-S3 and S5-S8. Figure S10-17. All differentially regulated PCG networks (STRING analysis), enriched Biological Process (GO), Molecular Function (GO), Cellular Component (GO), and KEGG Pathways in liver (Figure S10), duodenum (Figure S11), jejunum (Figure S12), ileum (Figure S13), colon (Figure S14), BAT (Figure S15), WAT (Figure S16), and skeletal muscle (Figure S17). Figure S18. RT-qPCR validation of selected lncRNAs from the RNA-Seq data. (PDF 143690 kb) [file 12864_2018_5235_MOESM2_ESM.pdf]
